# Osthole: A Coumarin with Dual Roles in Biology and Chemistry

**DOI:** 10.3390/biology14060588

**Published:** 2025-05-22

**Authors:** Min Lv, Haixia Ding, Hui Xu

**Affiliations:** 1 School of Marine Sciences, Ningbo University, Ningbo 315832, China; orgxuhui@nwsuaf.edu.cn; 2 College of Plant Protection, Northwest A&F University, Xianyang 712100, China; haixd8355@126.com

**Keywords:** osthole, osthole derivatives, biological activity, structural modification, mechanism of action, structure–activity relationship

## Abstract

Osthole is a natural coumarin-like compound isolated from the *Fructus cnidii*. In the last few years, this plant-derived product and its derivatives have aroused much attention for their interesting biological activities, including anticancer, anti-inflammatory, neuroprotective, and insecticidal effects. This review summarizes the recent progress on the biological activities of osthole and its derivatives from 2018 to early 2025, with a focus on their total synthesis, structural modifications, and mechanisms of action. Additionally, structure–activity relationships (SARs) of osthole derivatives are presented. This review aims to serve as a comprehensive reference for future research on osthole and its derivatives in both medicinal and agricultural applications.

## Simple Summary

Osthole is a natural coumarin-like compound isolated from various plant species. Osthole and its derivatives have received much attention due to their lots of interesting biological activities. In order to timely summarize their recent advances on biology and chemistry from 2018 to early 2025, here the biological activities, mechanisms of action, total synthesis, structural modifications, structure–activity relationships (SARs) of osthole and its derivatives are presented. Hopefully, this review can be used as the useful reference for researchers interested in the biology and chemistry of osthole derivatives for further in-depth research.

## 1. Introduction

**Figure 1:**
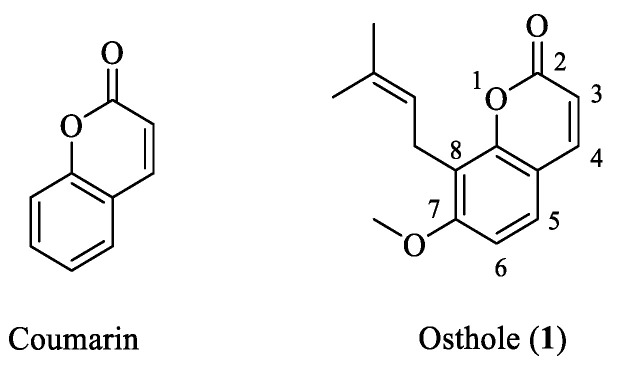
The chemical structures of coumarin and osthole (**1**).

Coumarin (Figure 1), which contains a 2 *H*-benzopyran-2-one core structure, is a naturally occurring compound with an aromatic odor that is widely distributed in various plant species. Vogel first isolated it from tonka beans (*Dipteryx odoranta* Wild; *Fabaceae* family) in 1820. Since then, thousands of natural coumarins have been isolated, described, and synthesized, and their biological activities have been studied from a variety of sources, including plants, bacteria, fungi, and chemical synthesis [1]. Osthole (**1**, Figure 1), often referred to as osthol (7-methoxy-8-(3-methyl-2-butenyl) coumarin), is a coumarin analog that was initially isolated from the *Cnidium* plant [2]. Osthole has shown many biological properties, including anticancer [3], anti-inflammatory [4], osteogenic [5], and neuroprotective activities, without significant toxic side effects [6]. It also has insecticidal activity in agriculture [7].

In this paper, recent advances on biological activities, total synthesis, and structural modifications of osthole and its derivatives from 2018 to early 2025 were reviewed. In addition, their structure–activity relationships (SARs) and mechanisms of action were presented for further in-depth research.

## 2. Bioactivities of Osthole and Its Derivatives

**Figure 2:**
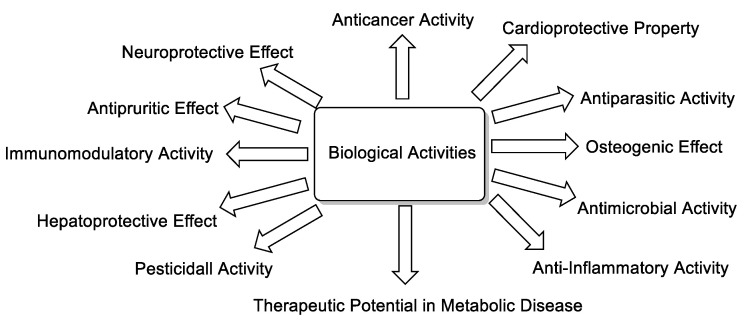
The biological activities of osthole and its derivatives.

The biological activities of osthole and its derivatives are outlined in Figure 2.

### 2.1. Anticancer Activity

Osthole has broad-spectrum anticancer activities against a range of tumor cells (Table 1), including endometrial carcinoma (EC) cells [8], breast cancer cells [9,10,11,12,13], ovarian carcinoma (OC) cells [14,15], colorectal (CRC) cancer cells [16,17,18,19,20], hepatocellular carcinoma (HCC) cells [21,22,23,24], bladder cancer cells [25], retinoblastoma (RB) cells [26], cervical cancer cells [27,28,29], head and neck squamous cell carcinoma (HNSCC) cells [30], glioma cells [31,32,33], gallbladder cancer (GBC) cells [34], lung cancer cells [35], human gastric cancer cells [36,37], esophageal squamous cell carcinoma (ESCC) cells [38], rhabdomyosarcoma (RMS) cells [39], pancreatic cancer cells [40], and nasopharyngeal cancer (NPC) cells [41]. Osthole can inhibit the PI3K/Akt signaling pathway in various cancer cells, such as in endometrial carcinoma, bladder cancer, retinoblastoma, cervical cancer, head and neck squamous cell carcinoma, and gastric cancer, which causes cancer cell proliferation, migration, invasion, and death [25,26,27,29,30,36]. Osthole has inhibited 4T1 cell proliferation through mTOR/SREBP1/FASN signaling, blocked cell entry into s phase, and induced apoptosis in breast cancer cells [11]. It can decrease the ASC/caspase-1/IL-1β inflammasome pathway and control colorectal cancer progression by regulating autophagy and mitochondrial signaling [17]. Osthole has inhibited glycolysis regulated by the GSK-3β/AMPK/mTOR pathway, increasing radiosensitivity of subcutaneously transplanted hepatocellular carcinoma in nude mice [21].

### 2.2. Immunomodulatory and Anti-Inflammatory Activities

Natural products offer several benefits, including the regulation of immune cell differentiation, mitigation of oxidative stress, and a reduction in inflammation. Osthole has acted on DSS-induced UC and LPS-induced ALI in mice by regulating p38 and JNK phosphorylation in the MAPK pathway [42]. Osthole has the potential to treat polycystic ovarian syndrome in mice by activating the Nrf2-Foxo1-GSH-NF-κB pathway [43]. Osthole can effectively prevent DSS-induced ulcerative colitis (UC) and LPS-induced acute lung injury (ALI) [42,44] and inhibit oleic acid/lipopolysaccharide-induced lipid accumulation and inflammatory response in hepatocytes via activating the PPARα signaling pathway [45,46]. Oshtole can treat rheumatoid arthritis (RA) and suppress knee osteoarthritis (KOA) by enhancing autophagy activated by the AMPK/ULK1 pathway [47,48,49,50]. Osthole significantly reduced skin damage and chronic itching associated with atopic dermatitis (AD) by increasing ZO-3 expression and blocking Akt phosphorylation in an AD mouse model [51,52,53,54], as well as having a protective effect against lipopolysaccharide-induced inflammation in BV2 cells [55,56]. Oshthole can ameliorate neurogenic and inflammatory nociceptive sensitization through the modulation of iNOS, COX-2, and inflammatory cytokines, and reduce the role of COX-2 in inflammation and ASD development [57,58].

### 2.3. Neuroprotective Effects

Osthole has had a neuroprotective impact on Alzheimer’s disease. Intranasal administration of osthole/borneols thermosensitive gel enhanced intracerebral bioavailability and reduced cognitive impairment in APP/PS1 transgenic mice [59,60,61,62]. Osthole can effectively reduce Parkinson’s disease symptoms by inhibiting JAK/STAT and MAPK pathways through attenuating 6-OHDA cytotoxicity in SH-SY5Y cells [63,64,65]. Osthole may delay neural stem cell (NSC) senescence through the p16-pRb signaling pathway [66]. Osthole provided protection against mechanical brain injury, effectively promoted cognitive dysfunction and neuronal recovery, and improved neurological function [67]. Osthole showed a substantial ability to lessen seizures [68]. Osthole reduced pain depression by inhibiting inflammation and oxidative stress-mediated neurotransmitter dysregulation [69].

### 2.4. Osteogenic Effects and Cardiovascular Benefits

Osthole may be used therapeutically to prevent osteoporosis [70]. Osthole has promoted osteogenic differentiation and inhibited osteoclastogenesis and osteoblast activity [71,72,73]. Osthole has been shown to have cardioprotective properties [74,75], and it can promote autophagy and protect rats from cardiac ischemia/reperfusion injury [76,77].

### 2.5. Hepatoprotective Effects and Therapeutic Potential in Metabolic Disease

Osthole has protected against D-Gal-induced liver injury through TLR4/MAPK/NF-κB pathways, and also prevented tamoxifen- and acetaminophen-induced liver injury [78,79,80]. Osthole has inhibited renal fibrosis through the IL-11/ERK1/2 signaling pathway, which is independent of TGF-β1 signaling [81,82]. Osthole has had the potential to help prevent and treat diabetic kidney disease (DKD) [83].

### 2.6. Antimicrobial and Antiparasitic Activities

Osthole has demonstrated inhibitory effects against a wide range of viruses, bacteria, and parasites. Natural products represent valuable sources of novel antimicrobial agents. Against *Listeria monocytogenes*, osthole strongly decreased Na^+^-K^+^-ATPase and Ca^2+^-Mg^2+^-ATPase activities, increased the production of reactive oxygen species (ROS), and disrupted the integrity and permeability of cell membranes [84,85,86]. Osthole has been successfully developed for its significant inhibitory effect on phytopathogenic fungi (Table 2) [87,88,89,90]. Osthole can effectively inhibit SARS-CoV-2 as well as tobacco mosaic virus (TMV) infection [91,92]. Furthermore, osthole demonstrated a high activity against *Rhipicephalus sanguineus* [93].

### 2.7. Insecticidal Activity

Osthole and its derivatives have shown pesticidal effects on *Mythimna separata*, *Myzus persicae*, *Tetranychus cinnabarinus*, and *Plutella xylostella* Linnaeus [94,95,96]. It can also be a strong attractant for oviposition on *Bactrocera dorsalis* [97]. Osthole had a significant lethal effect on the second instar larvae of *Spodoptera frugiperda* [98]. *Tetranychus urticae* Koch can be controlled by combining matrine and osthole in a certain ratio [99]; osthole had good contact and stomach poisoning effects on *Coptotermes formosanus*, *Sitophilus zeamais* Motschusky, and *Plutella xylostella* Linnaeus [100,101,102]. After treatment of osthole at 125 × 10^−3^ mg/mL for 24 h, the glutathione-*S*-transferase (GST) and acetylcholinesterase (AChE) activities in the *P. xylostella* larvae were significantly reduced. Furthermore, osthole showed larvicidal activity against mosquitoes [7,103].

### 2.8. Other Activities

Osthole can exert antipruritic effects by inhibiting or desensitizing cation channels TRPV1 and TRPV3 [104,105]. Osthole inhibited phosphodiesterase 4D activity, making it a promising bronchodilator for asthma treatment without targeting β2-adrenoceptors [106,107]. Osthole can also dramatically lower blood pressure by inhibiting the vascular constriction mediated by angiotensin II and CaCl_2_ [108].

## 3. Total Synthesis of Osthole and Its Derivatives

**Scheme 1:**
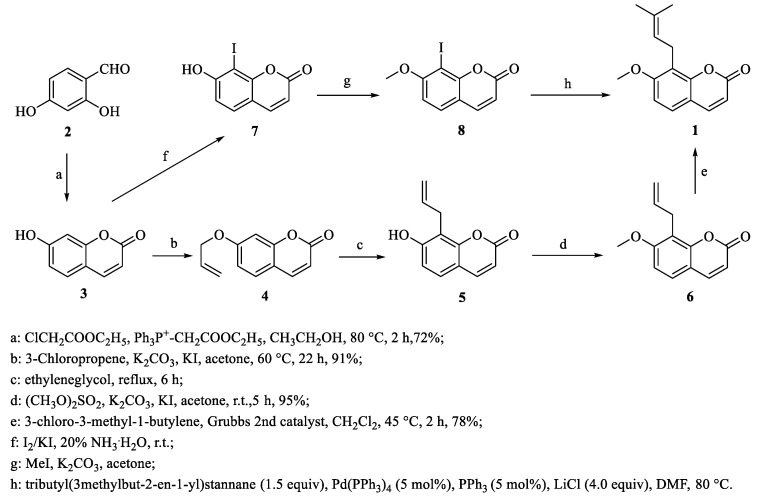
Synthesis of osthole (**1**) by Liu et al. and Yin et al. [109,110].

As described in Scheme 1, Liu et al. reported the synthesis of osthole (**1**) by 8-allyl-7-methoxycoumarin olefin complexation using 2,4 dihydroxybenzaldehyde (**2**) as a raw material [109]. Compound **3** was formed by reacting compound **2** with ethyl 2-chloroacetate. Subsequently, in the presence of potassium carbonate and potassium iodide, compound **3** was reacted with 3-chloropropene to afford compound **4**, which was then C-8 Claisen rearranged to give compound **5**. Compound **5** was methylated with dimethyl sulfate in acetone to give compound **6**. Ultimately, compound **1** was created with a 77.6% yield by reacting compound **6** with 3-chloro-3-methyl-1-butylene. Meanwhile, Yin et al. developed a palladium-catalyzed coupling reaction of tributyl (3-methylbut-2-en-1-yl) stannane with 7-methoxy-8-iodocoumarin (**8**), which was from 7-hydroxy-8-iodocoumarin (**7**), to synthesize compound **1** [110].

**Scheme 2:**
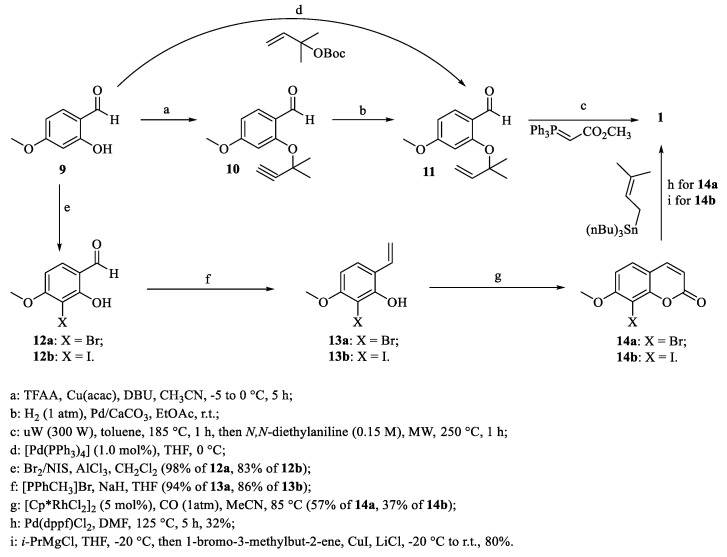
Synthesis of osthole (**1**) by Konrádová et al., Schmidt et al., Gulías et al., and Liu et al. [7,111,112,113].

The use of a microwave-promoted tandem Claisen rearrangement/olefin/cyclization method was an efficient approach for the synthesis of compound **1**. As shown in Scheme 2, Konrádová et al. prepared compound **1** from an aldehyde synthon (**9**) by substitution and reduction reactions at a 78% yield [111]. Schmidt et al. obtained compound **11** straight from compound **9** by substitution reaction, skipping the reduction step from compound **10** to compound **11**, and the yield of compound **1** was 59% [112]. Bromination or iodination of 2-hydroxy-4-methoxybenzaldehyde (**9**) with Br_2_ or NIS in the presence of aluminum chloride produced compounds **12a** and **12b**. These compounds were then reacted with the Wittig reagent to provide compounds **13a** and **13b**. The C-H activation/cyclization of compounds **13a** and **13b** was catalyzed by rhodium (III) to give coumarins **14a** and **14b**, respectively. Finally, compound **1** was synthesized from compound **14a** by Stille cross-coupling reaction [113]. Compared to palladium and nickel reagents, Grignard reagents align better with sustainable development principles. They can be modified into highly active derivatives by incorporating inexpensive and environmentally friendly transition metals, such as iron, zinc, and copper. Liu et al. therefore employed a non-precious metal-assisted Grignard reagent (using CuI and LiCl as promoters) to synthesize compound **1** from compound **14b** at an 80% yield. [7].

**Scheme 3:**
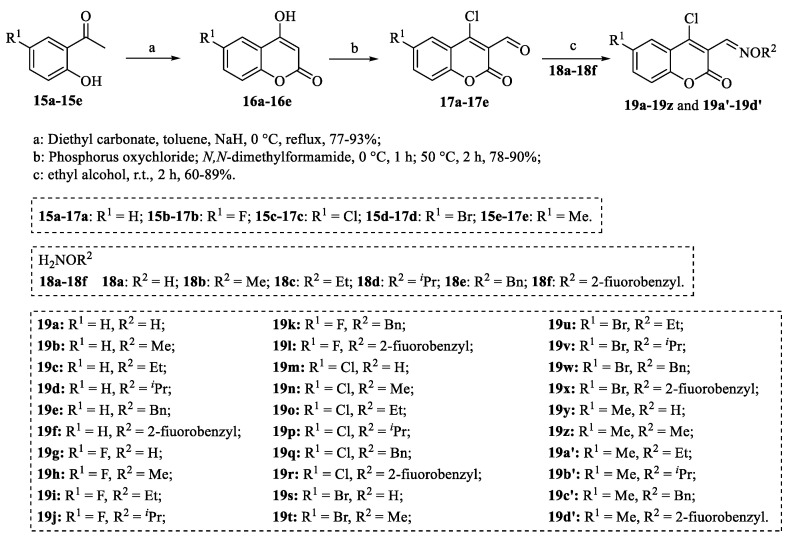
Synthesis of osthole derivatives **19a**–**19z** and **19a’**–**19d’**.

As shown in Scheme 3, a series of oxime derivatives of (*E*)-4-chloro-2-oxo-2*H*-chromene-3-carbaldehyde (**19a**–**19z** and **19a’**–**19d’**) were prepared through the successive three-step reactions from 2-hydroxyacetophenones (**15a**–**15e**) [114]. Among them, target compounds **19a** (EC_50_: 8.06 µg/mL), **19g** (EC_50_: 4.75 µg/mL), and **19n** (EC_50_: 5.43 µg/mL) showed a slightly lower inhibitory effect against *Botrytis cinerea* than boscalid (EC_50_: 0.88 µg/mL). In addition, the majority of compounds exhibited similar inhibitory effectiveness to boscalid against *Alternaria solani*; in particular, compounds **19c** (EC_50_: 5.39 µg/mL) and **19g** (EC_50_: 5.80 µg/mL) showed greater inhibitory effects than boscalid (EC_50_: 13.60 µg/mL). Against *Alternaria alternata*, compound **19g** (EC_50_: 5.74 µg/mL) displayed comparable inhibitory activity to boscalid (EC_50_: 2.55 µg/mL).

**Scheme 4:**
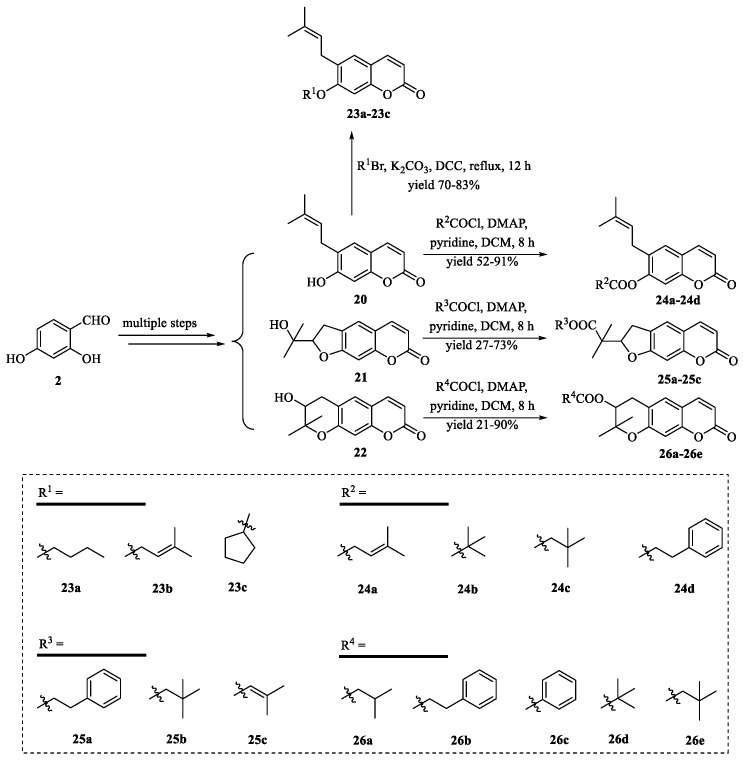
Synthesis of osthole derivatives **23a**–**23c**, **24a**–**24d**, **25a**–**25c**, and **26a**–**26e**.

As shown in Scheme 4, Ma et al. developed a series of coumarin derivatives using ring-merging and ring-opening strategies [115]. The intermediates **20**–**22** were synthesized in multistep reactions using 2,4-dihydroxybenzaldehyde (**2**) as the raw material. The treatment of compound **20** with the corresponding bromides provided compounds **23a**–**23c**. Acylation of **20**–**22** gave derivatives **24a**–**24d**, **25a**–**25c**, and **26a**–**26e**. Compound **24a** (EC_50_: 1.56 µg/mL) showed high inhibitory activity against *Pyricularia grisea*, which was more than 3 times more potent than chlorothalonil (EC_50_: 5.81 µg/mL). Compound **24a** showed good lethal activity against *Ostrinia nubilalis*, and its fatality rate was 67%, which was similar to that of matrine.

**Scheme 5:**
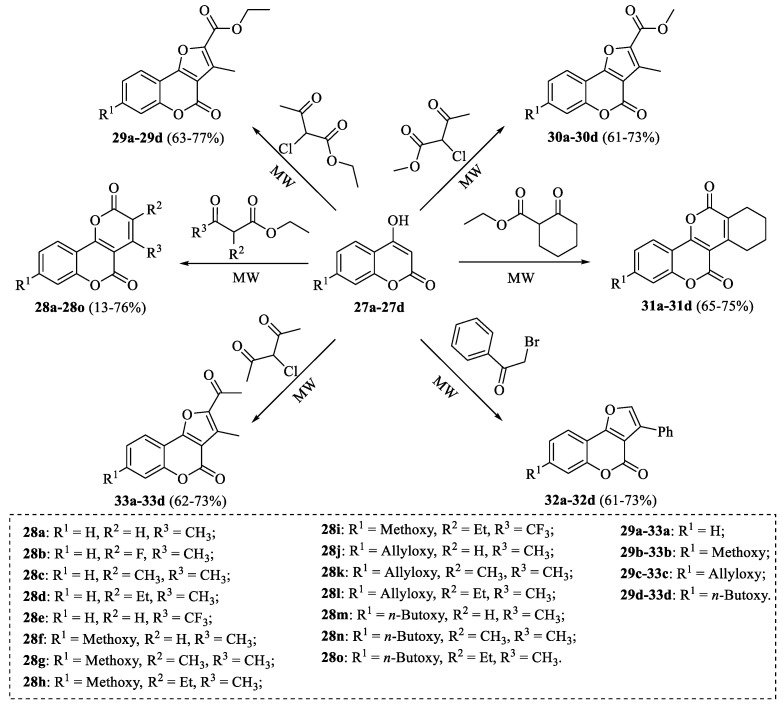
Synthesis of osthole derivatives **28**–**33**.

**Scheme 6:**
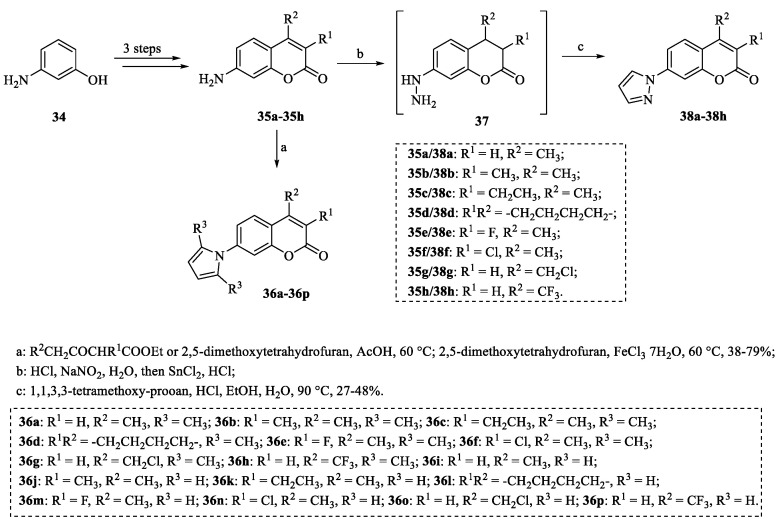
Synthesis of osthole derivatives **36a**–**36p** and **38a**–**38h**.

As shown in Scheme 5, a highly effective microwave-promoted technique was created for the synthesis of a number of pyrano [3,2-*c*]chromene-2,5-diones (**28a**–**28o**) and furo [3,2-*c*]coumarins (**29**(**a**–**d**)–**33**(**a**–**d**)) [116,117]. Among them, compounds **30c**, **32b**, **32c**, and **33b** showed high inhibitory activity against *B. cinerea*, *Colletotrichum capsica*, and *Rhizoctorzia solani*. In particular, compound **30c** was more effective than azoxystrobin (EC_50_: 0.158 µM) against *B. cinerea* (EC_50_: 0.110 µM) and *C. capsica* (EC_50_: 0.040 µM), making it the most promising choice. Additionally, compound **33b** (EC_50_: 0.051 µM) was equally as effective as the control azoxystrobin (EC_50_: 0.053 µM) against *R. solani*. As shown in Scheme 6, two series of 7-pyrazolecoumarins (**36a**–**36p**) and 7-pyrrolecoumarins (**38a**–**38h**) were synthesized from *m*-aminophenol (**34**) via the intermediates **35a**–**35h** and **37** [118]. In particular, compounds **36g**, **38a**–**d**, and **38h** showed good antifungal efficacy against *Cucumber anthrax* and *Alternaria* leaf spot, which was superior to the control osthole. Compared to osthole (EC_50_: 67.2 µg/mL), compound **36g** exhibited a greater inhibitory effect against *R. solani* (EC_50_: 15.4 µg/mL).

## 4. Structural Modifications of Osthole and Its Derivatives

**Figure 3:**
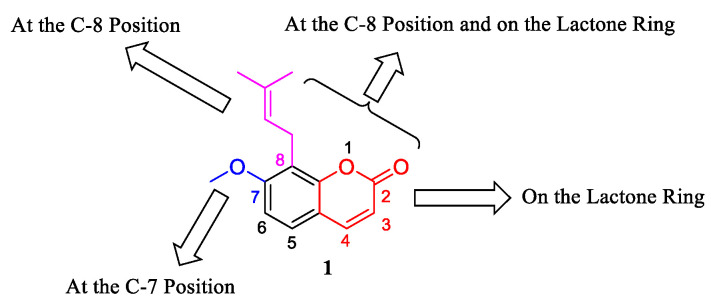
Structural modifications of osthole at different positions.

Structural modifications of osthole at different positions are outlined in Figure 3.

### 4.1. Structural Modifications on the Lactone Ring of Osthole

**Scheme 7:**
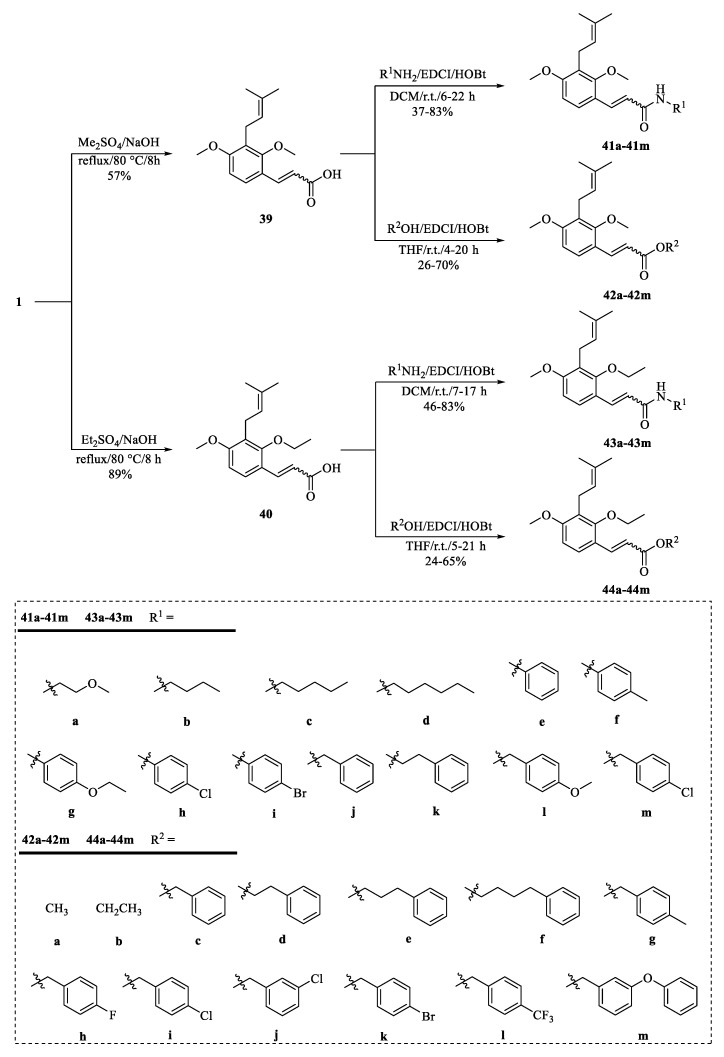
Synthesis of osthole derivatives **41**–**44**.

One of the key methods for finding novel insecticides is the structural modification of natural products (NPs). By opening the lactone of osthole (**1**), Xu et al. produced two series of amide/ester derivatives of osthole [95]. As seen in Scheme 7, compounds **41b**, **41l**, **42l**, **42m**, **44h**, **44l**, and **44m** showed more pronounced growth inhibition against *M. separata* than the precursor osthole, with corrected final mortality rates (CFMRs) of 62.0–68.9% at 1 mg/mL. The acaricidal activity of compounds **41b** (LC_50_: 0.238 mg/mL), **41i** (LC_50_: 0.226 mg/mL), and **42m** (LC_50_: 0.205 mg/mL) against *T. cinnabarinus* was 5.7–6.6 times higher than that of osthole (LC_50_: 1.359 mg/mL); thus, compounds **41b** and **42m** have potential for further development as pesticides.

**Scheme 8:**
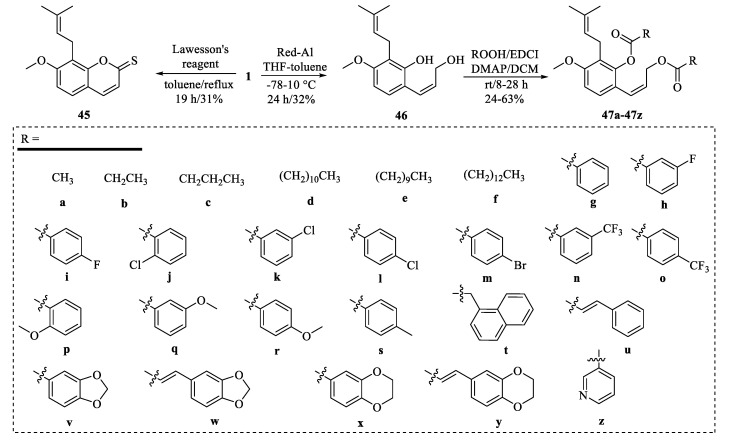
Synthesis of osthole derivatives **47a**–**47z**.

**Scheme 9:**
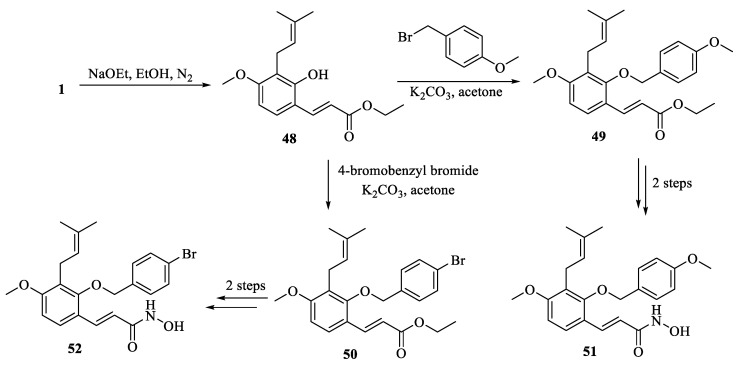
Synthesis of osthole derivatives **48**–**52**.

As shown in Scheme 8, a series of novel osthole-type ester derivatives (**47a**–**47z**) was prepared by modifying the osthole lactone ring [119]. Among them, compounds **47c** (LC_50_: 0.31 mg/mL), **47d** (LC_50_: 0.24 mg/mL), and **47e** (LC_50_: 0.28 mg/mL) showed strong acaricidal activity against *T. cinnabarinus*, as well as high control efficiency. In particular, compound **47d** had more than five times the acaricidal activity of precursor **1** (LC_50_: 1.22 mg/mL), and compounds **47i**, **47n**, and **47o** had 1.6–1.8 times the insecticidal activity against *M. separata* compared to **1**. Scheme 9 illustrates how *N*-hydroxyamide **51** was made by hydrolyzing **1** [120]. Compound **51** inhibited angiogenesis and suppressed tumor cell proliferation by targeting VEGFR2 signaling. In addition, HDAC6-induced Hsp90 hyperacetylation, and HDAC6-enhanced cyclin D1 and CDK4 degradation may be mediated by *N*-hydroxyamide **52**-induced G1 growth arrest [121].

### 4.2. Structural Modifications at the C-7 Position of Osthole

**Scheme 10:**
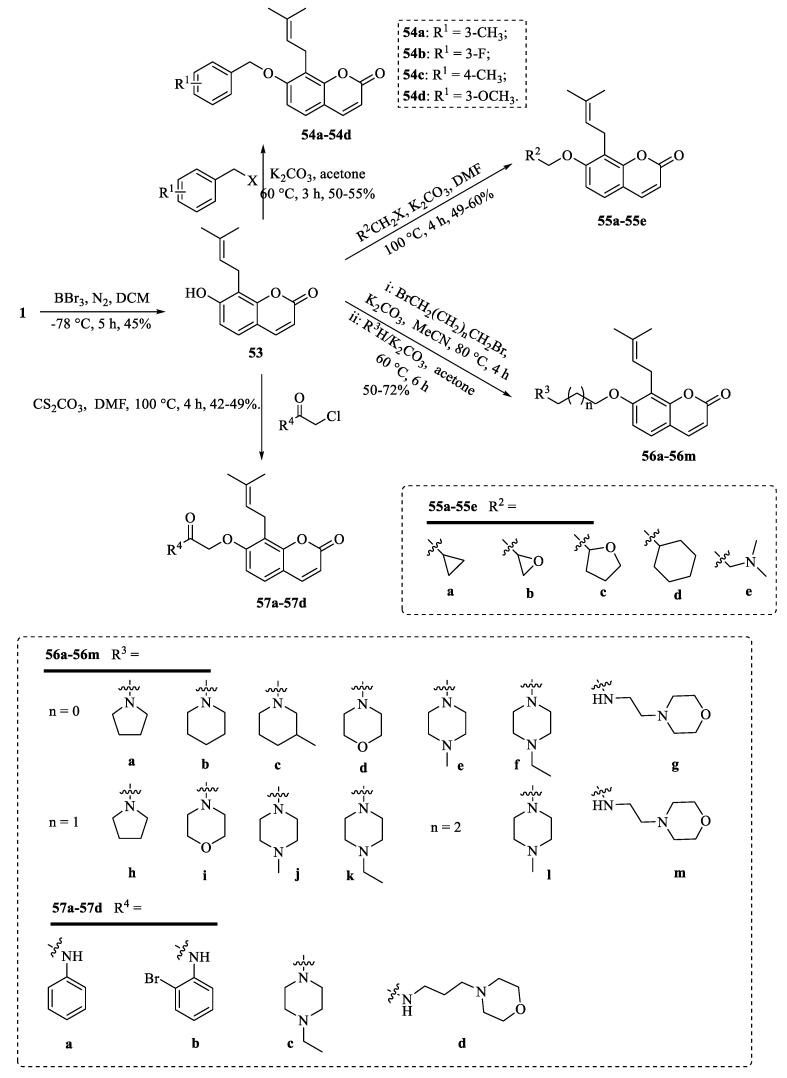
Synthesis of osthole derivatives **53**–**57**.

**Scheme 11:**
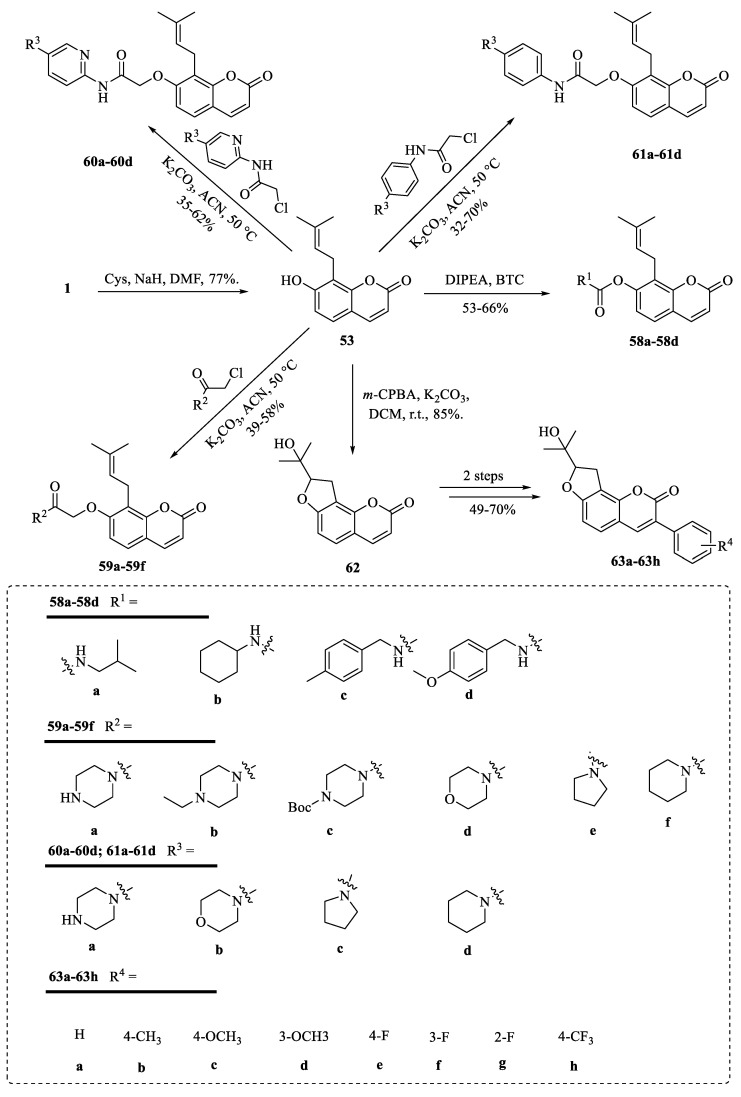
Synthesis of osthole derivatives **58**–**63**.

As depicted in Scheme 10, Zhou et al. prepared a series of osthole derivatives and tested their anti-inflammatory properties [42]. The majority of them were able to successfully suppress the release of inflammatory cytokines, including IL-6 in mouse macrophages J774A activated by LPS. Compound **56m** (IC_50_: 4.57 *μ*M) showed the strongest in vitro anti-inflammatory activity, which was 32 times higher than that of osthole. Compound **56m** significantly reduced DSS-induced ulcerative colitis and LPS-induced acute lung damage, and it is a potential anti-inflammatory candidate. As described in Scheme 11, Zhang et al. used an MTT assay to evaluate the cytoprotective ability of osthole-based compounds [122]. At 10 *μ*M, compound **59c** demonstrated significant neuroprotective efficacy against H_2_O_2_ (45.7 ± 5.5%), oxygen glucose deprivation (64.6 ± 4.8%), and Aβ_42_ (61.4 ± 5.2%).

The ability of compound **60c** (50.4 ± 7.1%) to suppress NO has outperformed that of the positive medication indomethacin. The neuroprotective effect of osthole may be improved by the addition of piperazine, tetrahydropyrrole, and aromatic amine groups.

### 4.3. Structural Modifications at the C-8 Position of Osthole

**Scheme 12:**
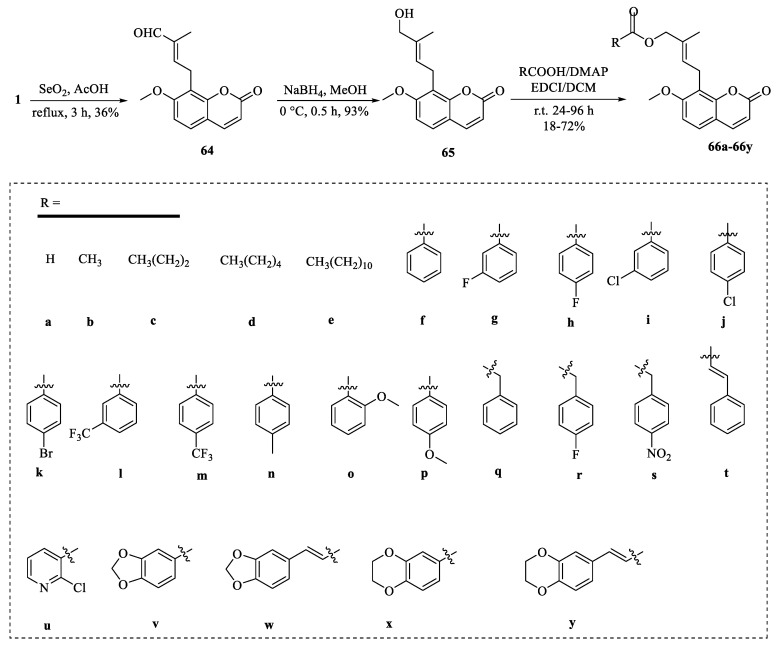
Synthesis of osthole-type ester derivatives **66a**–**66y**.

**Scheme 13:**
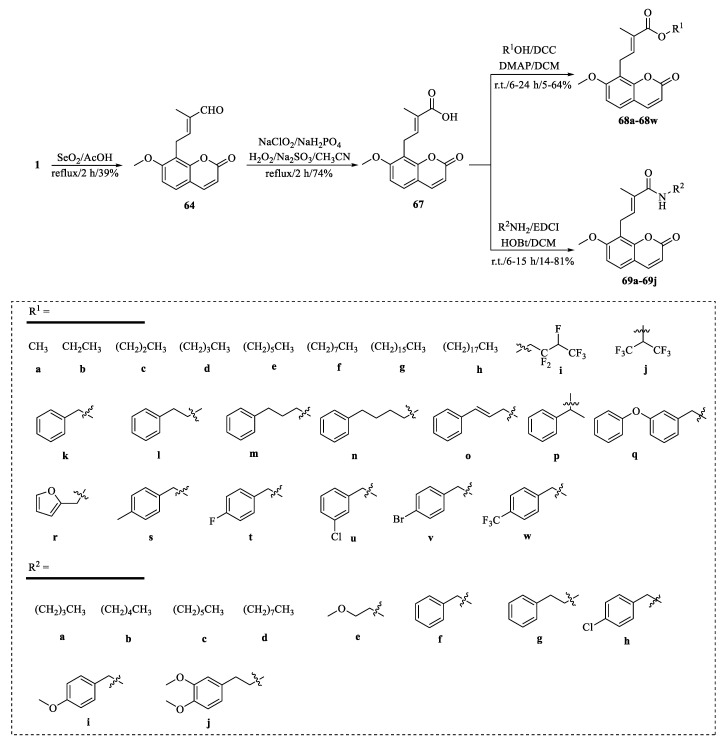
Synthesis of osthole derivatives **68a**–**68w** and **69a**–**69j**.

The carboxylic acid ester scaffold, as a potent pharmacophore, is widely utilized in structural modification strategies [123]. As depicted in Scheme 12, Hao et al. designed and synthesized a series of osthole ester derivatives (**66a**–**66y**) and evaluated their agricultural bioactivities [124]. Compound **66n** exhibited significant insecticidal activity against *M. separata* as compared to that of precursor **1**. Compounds **66i** and **66j** demonstrated 2.6 and 3.3 times the acaricidal activity of **1**, and good control effects in the glasshouse. As shown in Scheme 13, Hao et al. prepared two series of novel ester and amide derivatives modifying the C-4′ position of osthole (**68a**–**68w** and **69a**–**69j**) [125]. Compounds **68b** (LC_50_: 0.492 mg/mL) and **68e** (LC_50_: 0.509 mg/mL) showed 3.2 and 3.1 times more acaricidal activity than **1** (LC_50_: 1.578 mg/mL) against *T. cinnabarinus*, and the aphidicidal activities of compounds **68w** (LC_50_: 0.039 mg/mL), **69f** (LC_50_: 0.034 mg/mL), and **69g** (LC_50_: 0.038 mg/mL) against *Aphis citricola* were 1.9–2.1 times that of **1** (LC_50_: 0.073 mg/mL).

**Scheme 14:**
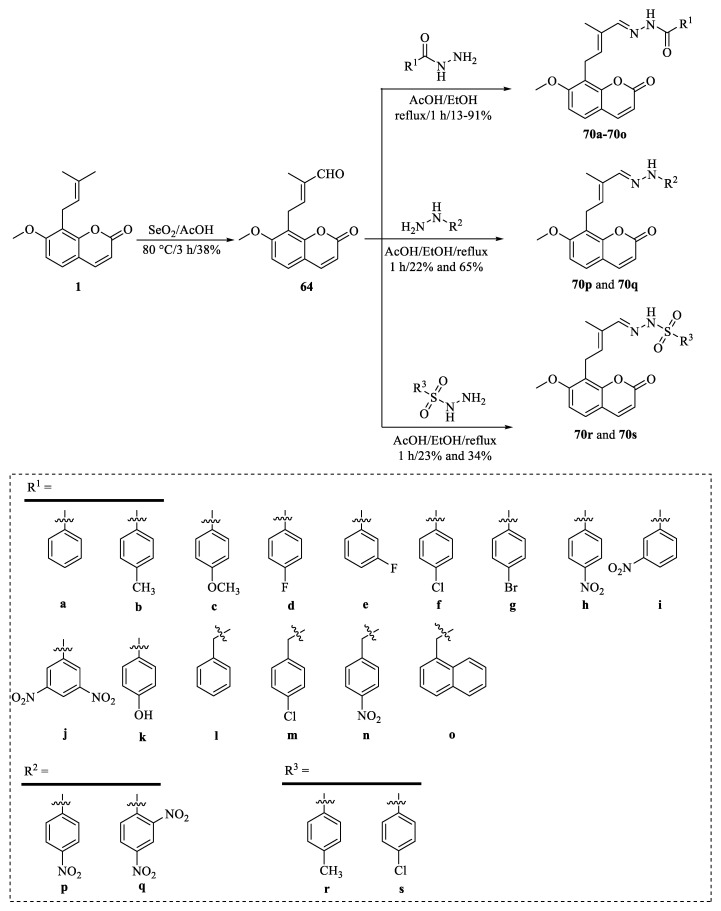
Synthesis of osthole derivatives **70a**–**70s**.

**Scheme 15:**
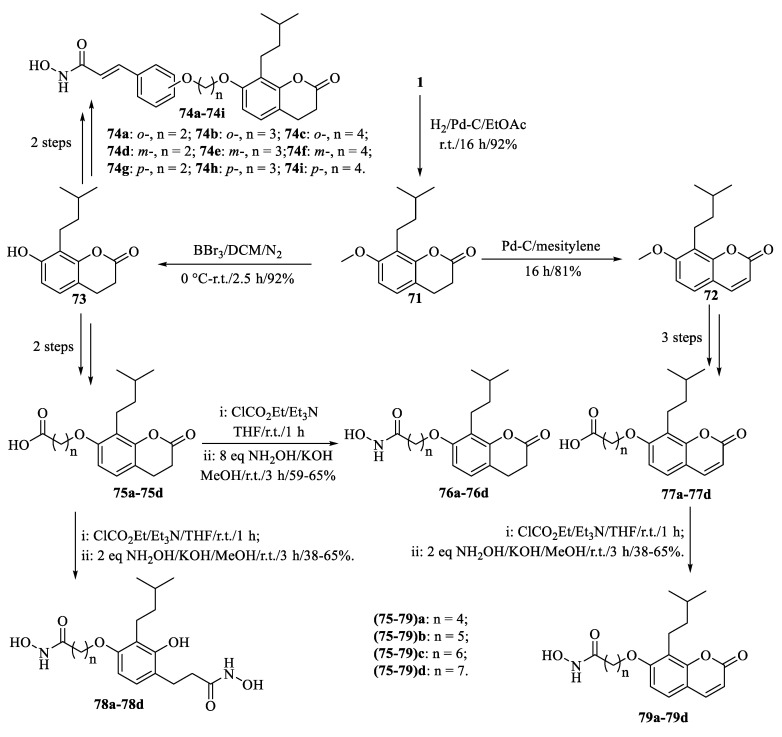
Synthesis of osthole derivatives **71**–**79**.

As shown in Scheme 14, Ren et al. regioselectively semi-prepared series of novel derivatives (**70a**–**70s**) containing hydrazone/acylhydrazone/sulfonylhydrazone skeletons at the C-8 position of osthole [126]. Benzoylhydrazone **70b** demonstrated 1.6 times the insecticidal activity of **1** against *M. separata*. The insecticidal activity can be enhanced by adding acylhydrazones to the 3′-methyl-2′-butylenyl fragment at the C-8 position of the osthole. As shown in Scheme 15, a number of osthole-based *N*-hydroxycinnamates (**71**–**79**) were prepared and examined for their ability to inhibit enzymes [127]. Comparable to that of suberoylanilide hydroxamic acid (SAHA; IC_50_: 24.5 nM), compounds **74d** (IC_50_: 24.5 nM), **74e** (IC_50_: 20.0 nM), and **74g** (IC_50_: 19.6 nM) showed good inhibitory effects against nuclear HDACs in HeLa cells. Furthermore, three different kinds of osthole derivatives were combined with SAHA-like hydroxamates. Compounds **78c**, **78d**, and **78c** had greater anti-HDAC-8 efficacy in addition to SAHA-like activity against HDAC-1, HDAC-4, and HDAC-6 [128].

**Scheme 16:**
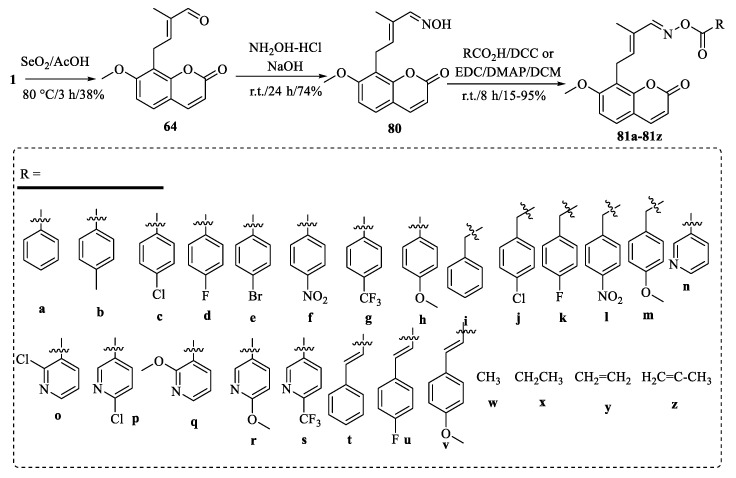
Synthesis of osthole oxime ester derivatives **81a**–**81z**.

**Scheme 17:**
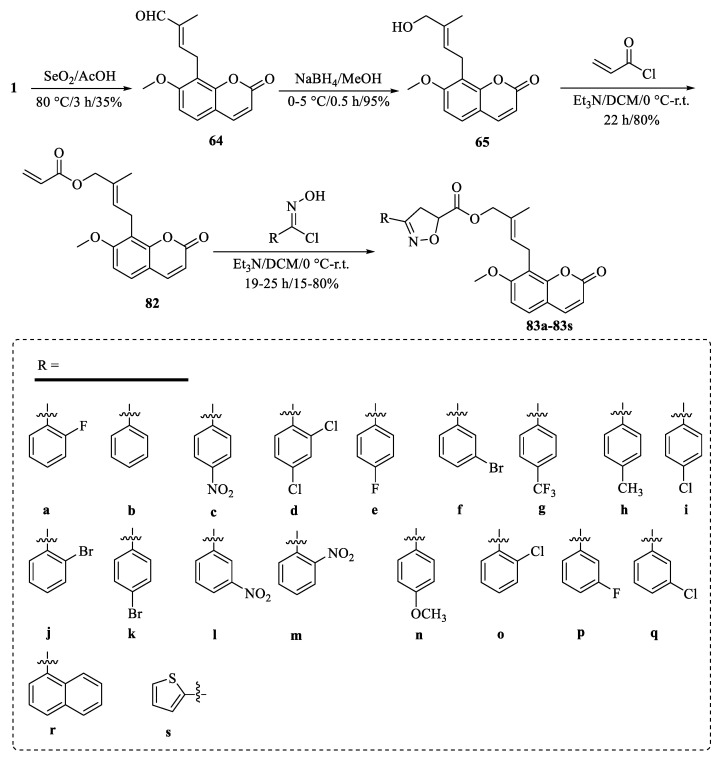
Synthesis of osthole derivatives **83a**–**83s**.

**Scheme 18:**
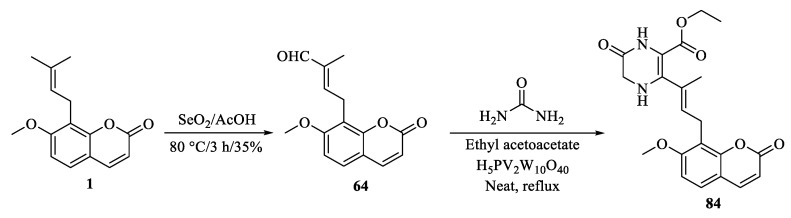
Synthesis of osthole derivative **84**.

A series of oxime ester derivatives of osthole (**81a**–**81z**, Scheme 16) were synthesized by selectively oxidizing **1** in the presence of selenium dioxide [129]. The acaricidal activity of compound **81c** (LC_50_: 0.316 mg/mL) against *T. cinnabarinus* was more than three times that of its precursor **1** (LC_50_: 1.011 mg/mL), and it was particularly effective in the greenhouse. Among them, compounds **81c** and **81f** had the strongest acaricidal activity against *M. separata*, with final mortality rates of 70.4% and 70.4%, respectively. Scheme 17 illustrated the construction of novel osthole esters (**83a**–**83s**) containing the isoxazoline fragment [130]. Compounds **82** (LC_50_: 0.75 mg/mL) and **83h** (LC_50_: 0.76 mg/mL) showed more than 1.5 times the acaricidal activity against *T. cinnabarinus* than **1** (LC_50_: 1.14 mg/mL), and also had good control in the greenhouse. At 1 mg/mL, compounds **83a** (FMR: 55.1%), **83c** (FMR: 62.0%), and **83o** (FMR: 55.1%) showed 1.5–1.6 times the growth inhibition against *M. separata* compared to **1**. The addition of the acryloyloxy group to the C-4′ position of osthole was critical for insecticidal and acaricidal properties. As shown in Scheme 18, the new dihydropyrimidinone analog of osthole (**84**) was synthesized by the Biginelli reaction [131]. Compound **84** exhibited potent inhibitory effects on colon (Colo-205), prostate (PC-3), leukemia (THP-1), and lung (A549) cancer cell lines, as well as on normal cell line fR-2, with IC_50_ values of 37.6, 40.8, 65.8, 67.8, and 68.8 µM, respectively.

### 4.4. Structural Modifications at the C-7 and C-8 Positions and on the Lactone Ring of Osthole

**Scheme 19:**
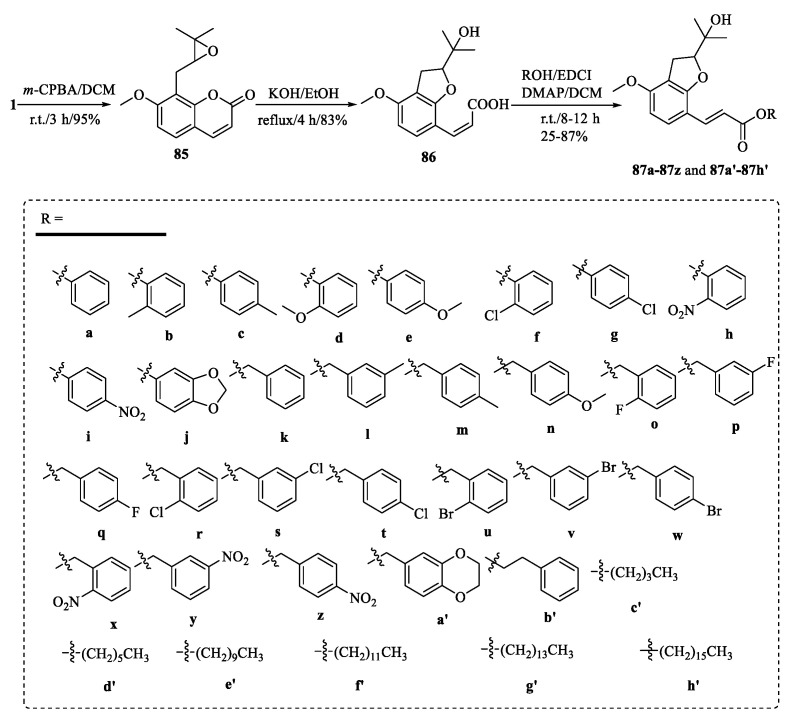
Synthesis of osthole derivatives **87a**–**87z** and **87a’**–**87h’**.

**Scheme 20:**
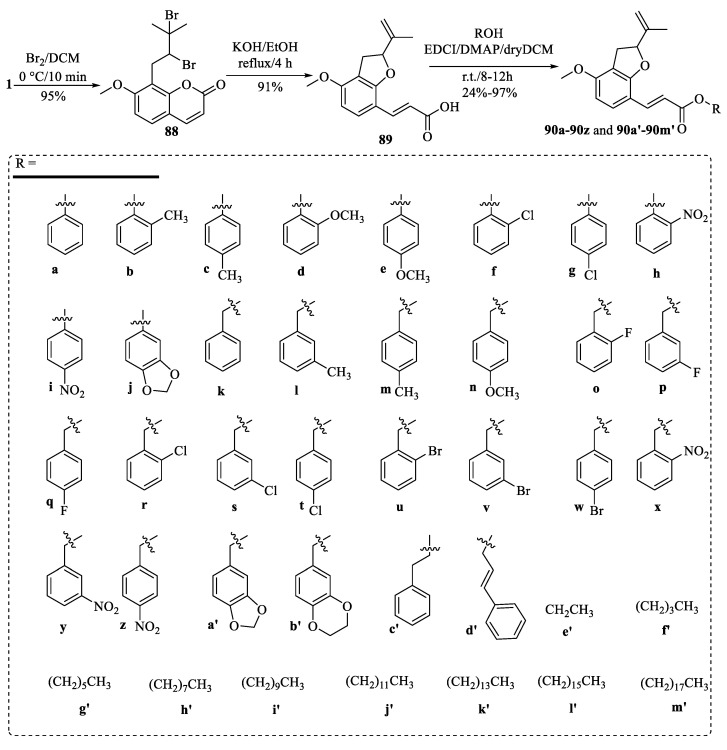
Synthesis of osthole derivatives **90a**–**90z** and **90a’**–**90m’**.

**Scheme 21:**
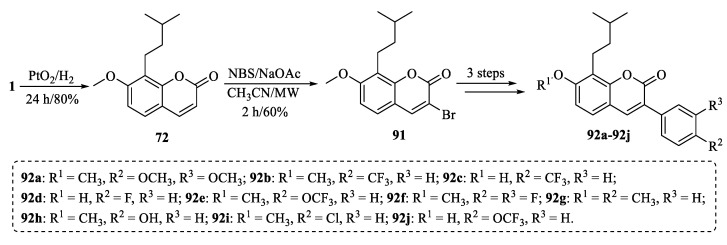
Synthesis of osthole derivatives **92a**–**92j**.

As described in Scheme 19, a series of new 2-isopropanol-4-methoxy-7-alkyl/aryloxycarbonyl-(*E*)-vinyl-2,3-dihydrobenzofuran derivatives (**87a**–**87z** and **87a’**–**87h’**) were designed and synthesized. Among all derivatives, compound **87b’** exhibited promising insecticidal activity against *P. xylostella* with an LC_50_ value of 0.759 mg/mL, which was higher than that of **1** (LC_50_: 1.415 mg/mL). Additionally, compound **87e’** displayed 3.3 times higher acaricidal activity and good control effects compared to those of **1** [94]. As shown in Scheme 20, Ren et al. used osthole as raw material to prepare a series of new acrylate derivatives (**90a**–**90z** and **90a’**–**90m’**) of isopropene-2,3-dihydrobenzofuran via consecutive bromination, rearrangement, and esterization processes [96]. Among them, the insecticidal activity of compound **90d’** (LC_50_: 0.368 mg/mL) against *P. xylostella* was 3.5 times greater than that of **1** (LC_50_: 1.281 mg/mL). The acaricidal activity of compound **90l’** (LC_50_: 0.165 mg/mL) was 8.3 times higher than that of **1** (LC_50_: 1.367 mg/mL) against *T. cinnabarinus*, and it could be further developed as an acaricide. As illustrated in Scheme 21, You et al. synthesized a series of 3-aryl-substituted derivatives (**92a**–**92j**) by structural modification at the C-7 and C-8 positions and on the lactone ring of osthole [132]. Some derivatives showed good inhibitory effects, among which compound **92e** inhibited MCF-7 and MDA-MB-231, with IC_50_ values of 0.24 and 0.31 µM, respectively, surpassing the parent compound osthole by over 100 times. The 7-methoxy and 3-aryl ring were critical for preserving cytotoxicity potency.

## 5. Structure–Activity Relationships of Osthole and Its Derivatives

**Figure 4:**
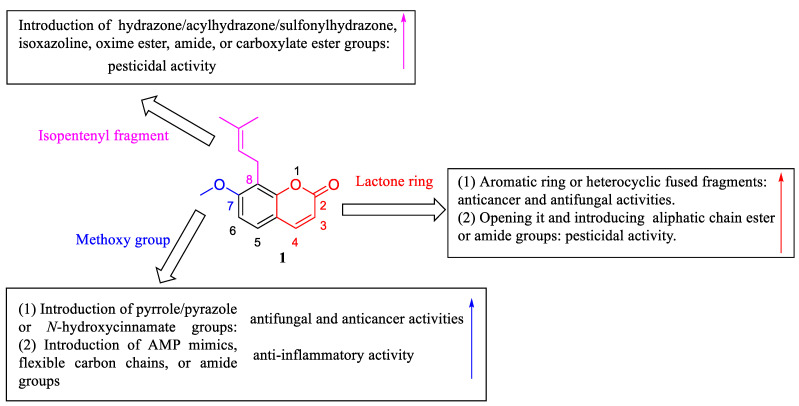
The structure–activity relationships of osthole and its derivatives.

As described in Figure 4, the structure–activity relationships (SARs) of osthole and its derivatives can be generally summarized as follows: (i) the introduction of an aromatic ring at the C-3 position of osthole can improve anticancer activity [120,121]. Incorporating heterocyclic fused fragments at the C-3 and C-4 positions resulted in derivatives showing good antifungal activity [116,117]. Opening the lactone ring and introducing aliphatic chain ester or amide groups were necessary for the insecticidal activity [94,95,119]. Additionally, the introduction of an *N*-hydroxylamide group can also augment anticancer activity [120,121]. (ii) The introduction of the pyrrole/pyrazole group at the C-7 position was necessary for the antifungal activity [118]. Adding the *N*-hydroxycinnamate group can also improve the anticancer activity [127]. Moreover, the introduction of AMP mimics, flexible carbon chains, and amide groups were beneficial for anti-inflammatory activity [42]. (iii) The isopentenyl fragment underwent oxidation and was subsequently converted into ester derivatives that exhibited inhibitory activity against acetylcholinesterase [62,124]. In particular, the introduction of *N*-benzoylthioureas/isoxazolines, hydrazones/acylhydrazones/sulfonylhydrazones, oxime esters, amides, and carboxylate esters groups were important for the pesticidal activity [125,126,131].

## 6. Conclusions

Recent advances in the biological activities, total synthesis, and structural modification of osthole and its derivatives were reviewed in the present work. Furthermore, the mechanisms of action and structure–activity relationships (SARs) of osthole and its derivatives were summarized. To find more pronounced compounds, extensive structural modification of osthole and its derivatives should be performed, and the scope of bioactivities can be broadened. With ongoing in-depth research on osthole and its derivatives, it is anticipated that the development of more osthole-type products will promote their practical applications in medicinal and agricultural fields, thereby contributing to human health and sustainable agricultural development.

## Figures and Tables

**Table 1:** The anticancer activity of osthole.

Tumor	Cell Lines/Model	Involved Mechanism	Period (h)	IC_50_ (µM)
Endometrial carcinoma	JEC, Ishikawa and KLE cells	Suppressing the proliferation and inducing apoptosis via inhibiting the PI3K/AKT signaling pathway	\	\ [8]
Breast cancer	4T1 cells	Inhibiting cell proliferation, blocking the cells from remaining in S-phase, and inducing apoptosis	48	178.40 [11]
	MDA-MB-231 cells	Inducing apoptosis arrest by elevation in the translation level of p53/Bax/caspase-3 p17 and downregulating of the Bcl-2 protein	\	\ [12]
	MDA-MB-231 cells BT-549 cells MDA-MB-468 cells MCF-7 cells	Inducing apoptosis and cell cycle arrest in TNBC cells through inhibition of STAT3 phosphorylation and nuclear translocation	24	129.40 [10]
				106.20 [10]
				105.40 [10]
				168.00 [10]
	MDA-MB-231 MDA-MB-231BO MCF-7 cells	Inhibiting cell migration and invasion via suppressing of ITGα3 and ITGβ5 signaling	24	29.8 [9]
				111.4 [9]
				260.6 [9]
	BT-474 and MCF-7 cells	Inducing cell cycle arrest, mitochondrial dysfunction, and ER stress	\	\ [13]
Ovarian carcinoma	A2780 cell OVCAR3 cell	Inducing LC3-mediated autophagy and GSDME-dependent pyroptosis	24	75.24 [15]
				73.58 [15]
	ES2 and OV90 cells	Targeting PI3K/MAPK signaling pathway	\	\ [14]
Colorectal cancer	HCT116 and HT29 cells	Regulating autophagy- and mitochondria-mediated signal transduction.	\	\ [17]
	HT-29 cells	Inducing apoptosis via cycle arrest and activating the intrinsic apoptotic pathway	24	115.08 [18]
	HCT116 and SW480 cells	Inhibits malignant phenotypes and inducing ferroptosis in KRAS mutant cells via suppressing AMPK/Akt signaling	\	\ [19]
	CCL-222 cells	Reducing the Akt mRNA expression and the activity p38 MAPK-α	24	23.00 [16]
Colorectal cancer	HT-29 cells	Inducing apoptosis of the cells via endoplasmic reticulum stress and autophagy	\	\ [20]
Hepatocellular carcinoma	HCC-LM3 cells	Inhibiting GSK-3β/AMPK/mTOR pathway-controlled glycolysis	\	[21]
	AKT/c-Met-driven HCC mouse model	Delaying hepatocarcinogenesis by suppressing AKT/FASN axis and ERK phosphorylation	\	[22]
	Huh7 and HepG2 cells	Resensitizing CD133+ hepatocellular carcinoma cells to cisplatin treatment via PTEN/AKT pathway	\	[24]
	Hepa1–6 cells	Inhibiting angiogenesis in an orthotopic mouse model of HCC via the NF-κB/VEGF signaling pathway.	\	[23]
Bladder cancer	5637 cells 253 J cells	Inhibiting invasion, migration, and epithelial–mesenchymal transition by inhibiting PI3K-AKT and JAK/STAT3 pathways	24	146.40 [25] 160.80 [25]
Retinoblastoma	Y-79 cells	Inhibiting the PI3K/AKT/mTOR pathway via regulating the hsa_circ_0007534/miR-214-3p axis	24	200.00 [26]
Cervical cancer	Hela B cells	Targeting DCLK1 mechanistically via interacting with Val468.	\	\ [28]
	CDDP-resistant cervical cancer cells	Reversing chemoresistance of CDDP-resistant cervical cancer to CDDP through repressing NRF2 expression partly associated with PI3K/AKT blockage	\	\ [29]
	HeLa, SiHa, C-33A and CaSki cells	Suppressing ATM/NF-κB signaling	\	\ [27]
Head and neck squamous cell carcinoma	FaDu cells Cal27 cells SCC25 cells HN4 cells	Inducing cell cycle arrest and apoptosis via suppressing the PI3K/AKT signaling pathway	24	122.35 [30] 183.32 [30] 189.26 [30] 237.42 [30]
Glioma	U87 and C6 cells	Triggering glioma cell necroptosis via ROS overproduction	\	\ [31]
	MOGGCCM and T98G cells	Inhibiting of anti-apoptotic Bcl-2 expression, decreasing mitochondrial membrane potential, and activation of caspase 3 via the mitochondrial (internal) pathway	\	\ [32]
	AA and GBM cells	Increasing the level of apoptosis via blocking of the expression of Bcl-2 and PI3K	\	\ [33]
Gallbladder cancer	NOZ and SGC-996 cells	Inhibiting the progression of human gallbladder cancer cells via the JAK/STAT3 signal pathway	\	\ [34]
Lung cancer	A549 cells	Inducing the apoptosis in A549 cells by inhibiting the histone deacetylase	24	188.50 [35]
Gastric cancer	HGC-27 and SGC-7901 cells	Inducting cell cycle arrest at G2/M phase by the regulation of PI3K/AKT	\	\ [36]
	N87 and SK-BR-3 cells	Inducing apoptosis and inhibiting AKT/MAPK pathway	\	\ [37]
Esophageal squamous cell carcinoma	KYSE150 cells KYSE410 cells	Inhibiting the PI3K/AKT signaling pathway via activation of PTEN	24	198.45 [38] 235.67 [38]
Rhabdomyosarcoma	TE671 cells	Inhibiting of cell cycle progression and inducing of apoptosis via the AKT and ERK kinase signaling pathways	96	4.05 * [39]
Pancreatic cancer	Panc 02 cells and RAW 264.7 cells	Attenuating tumor-infiltrating M2 macrophages	\	\ [40]
Nasopharyngeal cancer	NPC-039 and NPC-BM cells	Inducing apoptosis mediated by the Fas-Fas ligand and mitochondrial pathway	\	\ [41]

* µg/mL.

**Table 2:** Antibacterial and antifungal activities of osthole.

Object	Period (h)	Values (µg/mL)
*Listeria monocytogenes* ATCC19115	6	MIC = 62.5 [85]
*Staphylococcus aureus* ATCC25923	20–24	MIC = 128 [84]
*Escherichia coli* ATCC25922		MIC = 128 [84]
*Staphylococcus aureus* (MRSA) ATCC33591		MIC = 128 [84]
*Staphylococcus aureus* (Methicillin-resistant isolate) ATCC43300		MIC = 128 [84]
*Erwinia carotorora*	48	98.00% ^1^ [84]
*Ralstonia solanacearum*		25.00% ^1^ [84]
*Pseudomonas syringae pv. actinidiae*		68.00% ^1^ [84]
*Fusarium oxysporum*	72	57.40% ^2^ [86]
*Fusarium moniliforme* J. Sheld		70.66% ^2^ [86]
*Thanatephorus cucumeris* Donk		75.90% ^2^ [86]
*Penicillium choerospondiatis*	144	EC_50_ = 9.86 [89]
*Ustilaginoidea virens*	240	EC_50_ = 5.04 [88]
*Fusarium oxysporum*	168	EC_50_ = 6.40 [87]
*Cercospora sorghi*	72	EC_50_ = 1.46 [90]

^1^ At 0.1 mg/mL; ^2^ At 5 mg/mL.

## Data Availability

Data are contained within the article.

## Abbreviations

Akt	Protein kinase B
AMP	Adenosine 5′-monophosphate
AMPK	Adenosine 5′-monophosphate-activated protein kinase
ASD	Autistic spectrum disorders
COX-2	Cyclooxygenase 2
CREB	cAMP-response element binding protein
ERK1/2	Phosphor-extracellular signalregulated kinase 1/2
FASN	Fatty acid synthase
HDAC	Histone deacetylase
HER2	Human epidermal growth factor receptor 2
MAPK	Mitogen-activated protein kinase
NOS	Nitric oxide synthase
JAK	Janus kinase
NF-κB	Nuclear factor kappa-B
PI3K	Phosphatidylinositol 3 kinase
PTEN	Phosphate and tension homology deleted on chromosome ten
ROS	Reactive oxygen species
STAT3	Signal transducer and activator of transcription 3

## References

[1] Annunziata F., Pinna C., Dallavalle S., Tamborini L., Pinto A. (2020). An overview of coumarin as a versatile and readily accessible scaffold with broad-ranging biological activities. Int. J. Mol. Sci..

[2] Stefanachi A., Leonetti F., Pisani L., Catto M., Carotti A. (2018). Coumarin: A natural, privileged and versatile scaffold for bioactive compounds. Molecules.

[3] Yang S.J., Dai W.L., Wang J.N., Zhang X.L., Zheng Y.T., Bi S.Y., Pang L.W., Ren T.Q., Yang Y., Sun Y. (2022). Osthole: An up-to-date review of its anticancer potential and mechanisms of action. Front. Pharmacol..

[4] Li S.H., Li M.Y., Yuan T.T., Wang G.W., Zeng J.B., Shi Z.M., Liu J.H., Su J.C. (2024). Osthole activates the cholinergic anti-inflammatory pathway via α7nAChR upregulation to alleviate inflammatory responses. Chem. Biodivers..

[5] Hu H.Y., Chen M., Dai G.Z., Du G.Q., Wang X.Z., He J., Zhao Y.F., Han D.P., Cao Y.L., Zheng Y.X. (2016). An inhibitory role of osthole in rat MSCs osteogenic differentiation and proliferation via Wnt/β-catenin and Erk1/2-MAPK pathways. Cell. Physiol. Biochem..

[6] Li S.H., Yan Y.H., Jiao Y.A., Gao Z., Xia Y., Kong L., Yao Y.J., Tao Z.Y., Song J., Yan Y.P. (2016). Neuroprotective effect of osthole on neuron synapses in an Alzheimer’s disease cell model via upregulation of microRNA-9. J. Mol. Neurosci..

[7] Liu M., Liu Y., Hua X.W., Wu C.C., Zhou S., Wang B.L., Li Z.M. (2015). Synthesis of osthole derivatives with grignard reagents and their larvicidal activities on mosquitoes. Chin. J. Chem..

[8] Liang L., Yang B., Wu Y.Y., Sun L. (2021). Osthole suppresses the proliferation and induces apoptosis via inhibiting the PI3K/AKT signaling pathway of endometrial cancer JEC cells. Exp. Ther. Med..

[9] Chen Y.Q., Song H.Y., Zhou Z.Y., Ma J., Luo Z.Y., Zhou Y., Wang J.Y., Liu S., Han X.H. (2022). Osthole inhibits the migration and invasion of highly metastatic breast cancer cells by suppressing ITGα3/ITGβ5 signaling. Acta Pharmacol. Sin..

[10] Dai X.X., Yin C.T., Zhang Y., Guo G.L., Zhao C.G., Wang O.C., Xiang Y.Q., Zhang X.H., Liang G. (2018). Osthole inhibits triple negative breast cancer cells by suppressing STAT3. J. Exp. Clin. Cancer Res..

[11] Li X.Y., Zhang C.L., Wu E.H., Han L., Deng X.L., Shi Z.F. (2023). UPLC-Q-TOF/MS-based metabolomics approach reveals osthole intervention in breast cancer 4T1 cells. Int. J. Mol. Sci..

[12] Liu N., Tian H., Zhang G.D., Sun N., Wang S.M. (2022). Effect of combined treatment with lobaplatin and osthole on inducing apoptosis and inhibiting proliferation in human breast cancer MDA-MB-231 cells. Med. Oncol..

[13] Park W., Park S., Song G., Lim W. (2019). Inhibitory effects of osthole on human breast cancer cell progression via induction of cell cycle arrest, mitochondrial dysfunction, and ER stress. Nutrients.

[14] Bae H., Lee J.Y., Song J., Song G., Lim W. (2021). Osthole interacts with an ER-mitochondria axis and facilitates tumor suppression in ovarian cancer. J. Cell. Physiol..

[15] Liang J., Zhou J.L., Xu Y.Q., Huang X.F., Wang X.F., Huang W.H., Li H. (2020). Osthole inhibits ovarian carcinoma cells through LC3-mediated autophagy and GSDME-dependent pyroptosis except for apoptosis. Eur. J. Pharmacol..

[16] Arslan A.K.K., Uzunhisarcikli E., Ökçesiz A., Eken A., Yerer M.B. (2021). Anticaner effects of coumarin compounds osthole and imperatorin, alone and in combination with 5-fluorouacil in colon carcinoma cells. Acta Pol. Pharm..

[17] Song J., Ham J., Park W., Song G., Lim W. (2024). Osthole impairs mitochondrial metabolism and the autophagic flux in colorectal cancer. Phytomedicine.

[18] Torshizi G.H., Tabrizi M.H., Karimi E., Younesi A., Larian Z. (2024). Designing nanostructured lipid carriers modified with folate-conjugated chitosan for targeted delivery of osthole to HT-29 colon cancer cells: Investigation of anticancer, antioxidant, and antibacterial activities. Cancer Nanotechnol..

[19] Zhou X.H., Kang J., Zhang L.L., Cheng Y. (2023). Osthole inhibits malignant phenotypes and induces ferroptosis in KRAS-mutant colorectal cancer cells via suppressing AMPK/Akt signaling. Cancer Chemother. Pharmacol..

[20] Zhou X.H., Kang J., Zhong Z.D., Cheng Y. (2021). Osthole induces apoptosis of the HT-29 cells via endoplasmic reticulum stress and autophagy. Oncol. Lett..

[21] Huang H., Xue J., Xie M.L., Xie T. (2024). Osthole inhibits GSK-3β/AMPK/mTOR pathway-controlled glycolysis and increases radiosensitivity of subcutaneous transplanted hepatocellular carcinoma in nude mice. Strahlenther. Onkol..

[22] Mo Y.S., Wu Y., Li X., Rao H., Tian X.X., Wu D.N., Qiu Z.P., Zheng G.H., Hu J.J. (2020). Osthole delays hepatocarcinogenesis in mice by suppressing AKT/FASN axis and ERK phosphorylation. Eur. J. Pharmacol..

[23] Yao F., Zhang L.R., Jiang G.R., Liu M., Liang G.Q., Yuan Q. (2018). Osthole attenuates angiogenesis in an orthotopic mouse model of hepatocellular carcinoma via the downregulation of nuclear factor-κB and vascular endothelial growth factor. Oncol. Lett..

[24] Ye J.F., Sun D., Yu Y., Yu J.H. (2020). Osthole resensitizes CD133^+^ hepatocellular carcinoma cells to cisplatin treatment via PTEN/AKT pathway. Aging.

[25] Jiang Y.Z., Zhang M.Z., Wang L., Zhang L., Ma M.H., Jing M.X., Li J.P., Song R.D., Zhang Y.Q., Yang Z.Z. (2023). Potential mechanisms of osthole against bladder cancer cells based on network pharmacology, molecular docking, and experimental validation. BMC Complement. Med. Ther..

[26] Lv X.F., Yang H.J., Zhong H., He L., Wang L. (2022). Osthole exhibits an antitumor effect in retinoblastoma through inhibiting the PI3K/AKT/mTOR pathway via regulating the hsa_circ_0007534/miR-214-3p axis. Pharm. Biol..

[27] Che Y.L., Li J., Li Z.J., Li J., Wang S., Yan Y., Zou K., Zou L.J. (2018). Osthole enhances antitumor activity and irradiation sensitivity of cervical cancer cells by suppressing ATM/NF-κB signaling. Oncol. Rep..

[28] Jakubowicz-Gil J., Paduch R., Skalicka-Wozniak K., Sumorek-Wiadro J., Zajac A., Gawron A. (2019). Hsps responsible for apoptosis induction failure in cervical cancer cells upon osthole and tamoxifen treatment. Postępy Hig. Med. Doświadczalnej.

[29] Su J., Zhang F., Li X., Liu Z. (2019). Osthole promotes the suppressive effects of cisplatin on NRF2 expression to prevent drug-resistant cervical cancer progression. Biochem. Biophys. Res. Commun..

[30] Yang J., Zhu X.J., Jin M.Z., Cao Z.W., Ren Y.Y., Gu Z.W. (2020). Osthole induces cell cycle arrest and apoptosis in head and neck squamous cell carcinoma by suppressing the PI3K/AKT signaling pathway. Chem.-Biol. Interact..

[31] Huangfu M.J., Wei R.M., Wang J., Qin J.L., Yu D., Guan X., Li X.M., Fu M.L., Liu H.P., Chen X. (2021). Osthole induces necroptosis via ROS overproduction in glioma cells. FEBS Open Bio.

[32] Sumorek-Wiadro J., Zajac A., Badziul D., Langner E., Skalicka-Wozniak K., Maciejczyk A., Wertel I., Rzeski W., Jakubowicz-Gil J. (2020). Coumarins modulate the anti-glioma properties of temozolomide. Eur. J. Pharmacol..

[33] Sumorek-Wiadro J., Zajac A., Langner E., Skalicka-Wozniak K., Maciejczyk A., Rzeski W., Jakubowicz-Gil J. (2020). Antiglioma potential of coumarins combined with sorafenib. Molecules.

[34] Zou T.L., Wang H.F., Ren T., Shao Z.Y., Yuan R.Y., Gao Y., Zhang Y.J., Wang X.A., Liu Y.B. (2019). Osthole inhibits the progression of human gallbladder cancer cells through JAK/STAT3 signal pathway both in vitro and in vivo. Anti-Cancer Drugs.

[35] Abosharaf H.A., Diab T., Atlam F.M., Mohamed T.M. (2020). Osthole extracted from a citrus fruit that affects apoptosis on A549 cell line by histone deacetylasese inhibition (HDACs). Biotechnol. Rep..

[36] Xu X.J., Liu X.Y., Zhang Y. (2018). Osthole inhibits gastric cancer cell proliferation through regulation of PI3K/AKT. PLoS ONE.

[37] Yang Y., Ren F., Tian Z.Y., Song W., Cheng B.F., Feng Z.W. (2018). Osthole synergizes with HER2 inhibitor, trastuzumab in HER2-overexpressed N87 gastric cancer by inducing apoptosis and inhibition of AKT-MAPK pathway. Front. Pharmacol..

[38] Zhu X.B., Li Z.Z., Li T.T., Long F., Lv Y.S., Liu L., Liu X.F., Zhan Q.M. (2018). Osthole inhibits the PI3K/AKT signaling pathway via activation of PTEN and induces cell cycle arrest and apoptosis in esophageal squamous cell carcinoma. Biomed. Pharmacother..

[39] Jarzab A., Luszczki J., Guz M., Skalicka-Wozniak K., Halasa M., Smok-Kalwat J., Polberg K., Stepulak A. (2018). Combination of osthole and cisplatin against rhabdomyosarcoma TE671 cells yielded additive pharmacologic interaction by means of isobolographic analysis. Anticancer Res..

[40] Wang B.T., Zheng X., Liu J., Zhang Z., Qiu C.Y., Yang L., Zhang L.Q., Zhang Q., Gao H.W., Wang X.M. (2018). Osthole inhibits pancreatic cancer progression by directly exerting negative effects on cancer cells and attenuating tumor-infiltrating M2 macrophages. J. Pharmacol. Sci..

[41] Liu P.Y., Chang D.C., Lo Y.S., Hsi Y.T., Lin C.C., Chuang Y.C., Lin S.H., Hsieh M.J., Chen M.K. (2018). Osthole induces human nasopharyngeal cancer cells apoptosis through Fas-Fas ligand and mitochondrial pathway. Environ. Toxicol..

[42] Zhou Y., Du Z.T., Wu Q.Q., Guo M., Chen Z.C., Sun C.H., Li X.B., Zou Y., Zheng Z.W., Chen P. (2024). Discovery of novel osthole derivatives exerting anti-inflammatory effect on DSS-induced ulcerative colitis and LPS-induced acute lung injury in mice. Eur. J. Med. Chem..

[43] Jin S., Wang Y.S., Huang J.C., Wang T.T., Li B.Y., Guo B., Yue Z.P. (2024). Osthole exhibits the remedial potential for polycystic ovary syndrome mice through Nrf2-Foxo1-GSH-NF-κB pathway. Cell Biol. Int..

[44] Fan H.Y., Gan Z.F., Ji K., Li X., Wu J.B., Liu Y., Wang X.K., Liang H.Y., Liu Y.N., Li X.T. (2019). The in vitro and in vivo anti-inflammatory effect of osthole, the major natural coumarin from *Cnidium monnieri* (L.) Cuss, via the blocking of the activation of the NF-κB and MAPK/p38 pathways. Phytomedicine.

[45] Zhao X., Wang F., Zhou R.J., Zhu Z.Y., Xie M.L. (2018). PPARα/γ antagonists reverse the ameliorative effects of osthole on hepatic lipid metabolism and inflammatory response in steatohepatitic rats. Inflammopharmacology.

[46] Zhao X., Xue J., Xie M.L. (2019). Osthole inhibits oleic acid/lipopolysaccharide-induced lipid accumulation and inflammatory response through activating PPARα signaling pathway in cultured hepatocytes. Exp. Gerontol..

[47] Jiang X.L., Lu Z.J., Zhang Q., Yu J.L., Han D., Liu J.H., Li P., Li F. (2023). Osthole: A potential AMPK agonist that inhibits NLRP3 inflammasome activation by regulating mitochondrial homeostasis for combating rheumatoid arthritis. Phytomedicine.

[48] Lin X., Chen J., Tao C., Luo L., He J., Wang Q. (2023). Osthole regulates N6-methyladenosine-modified TGM2 to inhibit the progression of rheumatoid arthritis and associated interstitial lung disease. MedComm.

[49] Zhang W.L., Li Z.C., Dai Z.W., Chen S.Y., Guo W.X., Wang Z.W., Wei J.S. (2023). GelMA hydrogel as a promising delivery system for osthole in the treatment of rheumatoid arthritis: Targeting the miR-1224-3p/AGO1 axis. Int. J. Mol. Sci..

[50] Ma T., Wang X.P., Qu W.J., Yang L.S., Jing C., Zhu B.R., Zhang Y.K., Xie W.P. (2022). Osthole suppresses knee osteoarthritis development by enhancing autophagy activated via the AMPK/ULK1 pathway. Molecules.

[51] Fu X.P., Hong C.H. (2019). Osthole attenuates mouse atopic dermatitis by inhibiting thymic stromal lymphopoietin production from keratinocytes. Exp. Dermatol..

[52] Kordulewska N.K., Król-Grzymala A. (2024). The effect of osthole on transient receptor potential channels: A possible alternative therapy for atopic dermatitis. J. Inflamm. Res..

[53] Kordulewska N.K., Topa J., Stryinski R., Jarmolowska B. (2022). Osthole inhibits expression of genes associated with toll-like receptor 2 signaling pathway in an organotypic 3D skin model of human epidermis with atopic dermatitis. Cells.

[54] Zhou Y., Shi J.X., Qi M.X., Li X., Yang Y., Zhu C., Wang C.M., Tang Z.X., Ma Y.X., Yu G. (2023). Osthole relieves skin damage and inhibits chronic itch through modulation of Akt/ZO-3 pathway in atopic dermatitis. Eur. J. Pharmacol..

[55] Bao Y.X., Meng X.L., Liu F.N., Wang F., Yang J.H., Wang H.Y., Xie G.H. (2018). Protective effects of osthole against inflammation induced by lipopolysaccharide in BV2 cells. Mol. Med. Rep..

[56] Liu C.H., Chen M.Y., Kuo Y.H., Cheng J.C., Chang L.Z., Chang M.S., Chuang T.N., Hsieh W.T., Xiao Y.R., Wu B.T. (2023). Osthole antagonizes microglial activation in an NRF2-dependent manner. Molecules.

[57] Singh G., Bhatti R., Mannan R., Singh D., Kesavan A., Singh P. (2019). Osthole ameliorates neurogenic and inflammatory hyperalgesia by modulation of iNOS, COX-2, and inflammatory cytokines in mice. Inflammopharmacology.

[58] Kordulewska N.K., Kostyra E., Chwala B., Moszynska M., Cieslinska A., Fiedorowicz E., Jarmolowska B. (2019). A novel concept of immunological and allergy interactions in autism spectrum disorders: Molecular, anti-inflammatory effect of osthole. Int. Immunopharmacol..

[59] Chu Q.B., Zhu Y.F., Cao T.J., Zhang Y., Chang Z.C., Liu Y., Lu J.H., Zhang Y.Z. (2020). Studies on the neuroprotection of osthole on glutamate-induced apoptotic cells and an Alzheimer’s disease mouse model via modulation oxidative stress. Appl. Biochem. Biotechnol..

[60] Liu J.M., Wu Q.H., Wu Q.Q., Zhong G.C., Liang Y., Gu Y., Hu Y.H., Wang W.J., Hao N., Fang S.H. (2023). Modulating endoplasmic reticulum stress in APP/PS1 mice by Gomisin B and osthole in Bushen-Yizhi formula: Synergistic effects and therapeutic implications for Alzheimer’s disease. Phytomedicine.

[61] Wang X., Fu X.M., Luo X.R., Lai Y.Y., Cai C.P., Liao Y.F., Dai Z., Fang S.H., Fang J.S. (2024). Network proximity analysis deciphers the pharmacological mechanism of osthole against D-galactose induced cognitive disorder in rats. Molecules.

[62] Yu X., Zhang Y., Zhang M.J., Chen Y.F., Yang W.D. (2022). Natural products as sources of acetylcholinesterase inhibitors: Synthesis, biological activities, and molecular docking studies of osthole-based ester derivatives. Front. Plant Sci..

[63] Barangi S., Hosseinzadeh P., Karimi G., Tayarani-Najaran Z., Mehri S. (2023). Osthole attenuated cytotoxicity induced by 6-OHDA in SH-SY5Y cells through inhibition of JAK/STAT and MAPK pathways. Iran. J. Basic Med. Sci..

[64] Wang S.G., Ding S.L., Wang Y.L., He C.Y., Yao R.P., Jin H. (2023). Osthole exerts neuroprotection in a mouse model of Parkinson’s disease. Curr. Top. Nutraceutical Res..

[65] Wang Y., Zhou Y., Wang X., Zhen F., Chen R., Geng D.Q., Yao R.Q. (2019). Osthole alleviates MPTP-induced Parkinson’s disease mice by suppressing Notch signaling pathway. Int. J. Neurosci..

[66] Yao Y.J., Liang X.C., Shi Y., Lin Y., Yang J.X. (2018). Osthole delays tert-butyl hydroperoxide-induced premature senescence in neural stem cells. Cell. Reprogramm..

[67] Yan Y.H., Kong L., Xia Y., Liang W.B., Wang L.T., Song J., Yao Y.J., Lin Y., Yang J.X. (2018). Osthole promotes endogenous neural stem cell proliferation and improved neurological function through Notch signaling pathway in mice acute mechanical brain injury. Brain Behav. Immun..

[68] Zhao C., Rollo B., Shahid Javaid M., Huang Z., He W., Xu H., Kwan P., Zhang C. (2024). An integrated in vitro human iPSCs-derived neuron and in vivo animal approach for preclinical screening of anti-seizure compounds. J. Adv. Res..

[69] Singh L., Kaur A., Garg S., Bhatti R. (2021). Skimmetin/osthole mitigates pain-depression dyad via inhibiting inflammatory and oxidative stress-mediated neurotransmitter dysregulation. Metab. Brain Dis..

[70] Jin Z.X., Liao X.Y., Da W.W., Zhao Y.J., Li X.F., Tang D.Z. (2021). Osthole enhances the bone mass of senile osteoporosis and stimulates the expression of osteoprotegerin by activating β-catenin signaling. Stem Cell Res. Ther..

[71] Chen J.C., Liao X.F., Gan J.W. (2023). Review on the protective activity of osthole against the pathogenesis of osteoporosis. Front. Pharmacol..

[72] Ma Y., Wang L.N., Zheng S.Y., Xu J.K., Pan Y.L., Tu P.C., Sun J., Guo Y. (2019). Osthole inhibits osteoclasts formation and bone resorption by regulating NF-κB signaling and NFATc1 activations stimulated by RANKL. J. Cell. Biochem..

[73] Xu T.S., Yin J.Y., Dai X., Liu T.Y., Shi H.F., Zhang Y.Y., Wang S., Yue G.Y., Zhang Y.F., Zhao D.D. (2024). Cnidii Fructus: A traditional Chinese medicine herb and source of antiosteoporotic drugs. Phytomedicine.

[74] García-Arroyo F.E., Gonzaga-Sánchez G., Silva-Palacios A., Roldán F.J., Loredo-Mendoza M.L., Alvarez-Alvarez Y.Q., Coyotl J.A.D., Orozco K.A.V., Tapia E., Osorio-Alonso H. (2023). Osthole prevents heart damage induced by diet-induced metabolic syndrome: Role of fructokinase (KHK). Antioxidants.

[75] Li Y.L., Li Y.Q., Shi F.G., Wang L.N., Li L.S., Yang D.L. (2018). Osthole attenuates right ventricular remodeling via decreased myocardial apoptosis and inflammation in monocrotaline-induced rats. Eur. J. Pharmacol..

[76] Hou H., Yang Y.P., Chen R., Guo Z.P. (2023). Osthole protects H9c2 cardiomyocytes against trastuzumab-induced damage by enhancing autophagy through the p38MAPK/mTOR signaling pathway. Toxicol. In Vitro.

[77] Lu W.F., He Y., Yu T., Huang K.L., Liu S.Z. (2019). Osthole down-regulates miR-30a and promotes autophagy to protect rats against myocardial ischemia/reperfusion injury. Turk Gogus Kalp Damar..

[78] Cai Y., Sun W., Zhang X.X., Lin Y.D., Chen H., Li H. (2018). Osthole prevents acetaminophen-induced liver injury in mice. Acta Pharmacol. Sin..

[79] Ma Z., Peng L., Chu W.H., Wang P., Fu Y.Q. (2023). Osthole alleviates D-galactose-induced liver injury *in vivo* via the TLR4/MAPK/NF-κB pathways. Molecules.

[80] Zhou W.B., Zhang X.X., Cai Y., Sun W., Li H. (2019). Osthole prevents tamoxifen-induced liver injury in mice. Acta Pharmacol. Sin..

[81] Wu F., Zhao Y., Shao Q.Q., Fang K., Dong R.L., Jiang S.J., Lu F.E., Luo J.L., Chen G. (2021). Ameliorative effects of osthole on experimental renal fibrosis *in vivo* and *in vitro* by inhibiting IL-11/ERK1/2 signaling. Front. Pharmacol..

[82] Zhang S.P., Huang Q., Cai X.X., Jiang S., Xu N., Zhou Q., Cao X.Y., Hultström M., Tian J., Lai E.Y. (2018). Osthole ameliorates renal fibrosis in mice by suppressing fibroblast activation and epithelial-mesenchymal transition. Front. Physiol..

[83] Li Q.S., Wang Y.F., Yan J., Yuan R.Y., Zhang J.M., Guo X.H., Zhao M.M., Li F.F., Li X.T. (2024). Osthole ameliorates early diabetic kidney damage by suppressing oxidative stress, inflammation and inhibiting TGF-81/Smads signaling pathway. Int. Immunopharmacol..

[84] Guo Y.Y., Chen J.B., Ren D., Du B., Wu L., Zhang Y.Y., Wang Z.Y., Qian S. (2021). Synthesis of osthol-based botanical fungicides and their antifungal application in crop protection. Bioorg. Med. Chem..

[85] Kong Y., Yan H., Hu J.J., Dang Y.X., Han Z.H., Tian B., Wang P.X. (2024). Antibacterial activity and mechanism of action of osthole against *Listeria monocytogenes*. J. Agric. Food Chem..

[86] Zheng H.L., Chen Y.H., Guo Q.L., Wei H., Yue J.Y., Zhou H.Y., Zhao M.M. (2021). Inhibitory effect of osthole from *Cnidium monnieri* (L.) *Cusson* on *Fusarium oxysporum*, a common fungal pathogen of potato. Molecules.

[87] Hu K., Li R.Y., Mo F.X., Ding Y., Zhou A.A., Guo X., Li R.T., Li M., Ou M.G., Li M. (2023). Natural product osthole can significantly disrupt cell wall integrity and dynamic balance of *Fusarium oxysporum*. Pestic. Biochem. Physiol..

[88] Hu X.F., Wang J., Zhang Y.B., Li R.Y., Li M. (2023). Molecular mechanism of osthole against chitin synthesis of *Ustilaginoidea virens* based on combined transcriptome and metabolome analyses. Pestic. Biochem. Physiol..

[89] Lai D., Wang D.L., Shao X.H., Qin J., Zhuang Q.L., Xu H.H., Xiao W.Q. (2024). Comparative physiological and transcriptome analysis provide insights into the inhibitory effect of osthole on *Penicillium choerospondiatis*. Pestic. Biochem. Physiol..

[90] Sun Y.B., Chen Y., Liu T., Wang Y.Y., Wang Y., Han L.R., Ma Z.Q., Feng J.T. (2021). Evaluating the efficacy of osthole and matrine for control of Sorghum purple spot. J. Plant Dis. Prot..

[91] Yu C.Q., Xie T.T., Liu S.H., Bai L.G. (2024). Fabrication of a biochar-doped monolithic adsorbent and its application for the extraction and determination of coumarins from *Angelicae Pubescentis Radix*. J. Chromatogr. A.

[92] Chen Y.H., Guo D.S., Lu M.H., Yue J.Y., Liu Y., Shang C.M., An D.R., Zhao M.M. (2020). Inhibitory effect of osthole from *Cnidium monnieri* on tobacco mosaic virus (TMV) infection in *Nicotiana glutinosa*. Molecules.

[93] Ni J., Ren Q.Y., Luo J., Chen Z., Xu X.F., Guo J.H., Tan Y.C., Liu W.G., Qu Z.Q., Wu Z.G. (2020). Ultrasound-assisted extraction extracts from *Stemona japonica* (Blume) Miq. and *Cnidium monnieri* (L.) Cuss. could be used as potential *Rhipicephalus sanguineus* control agents. Exp. Parasitol..

[94] Ren Z.L., Lv M., Yang Y.L., Gu S.Y., Li L.L., Liu H.Q., Xu H. (2025). Structural optimization of natural plant products: Construction, pesticidal activities, and toxicology study of new 2-isopropanol-4-methoxy-7-alkyl/aryloxycarbonyl-(*E*)-vinyl-2,3-dihydrobenzofurans. J. Agric. Food Chem..

[95] Xu J.W., Lv M., Li T.Z., Wen H.P., Xu H. (2023). Optimization of osthole in the lactone ring as an agrochemical candidate: Synthesis, characterization, and pesticidal activities of osthole amide/ester derivatives and their effects on morphological changes of mite epidermis. J. Agric. Food Chem..

[96] Ren Z.L., Lv M., Liu H.Q., Wen H.P., Zhang Y.L., Xu H. (2023). Optimization of osthole as a pesticide candidate: Synthesis, crystal structures, and agrochemical properties of acrylate derivatives of isopropenyl 2,3-dihydrobenzofurans. J. Agric. Food Chem..

[97] Dong F., Chen X., Men X.Y., Li Z., Kong Y.J., Yuan Y.Y., Ge F. (2023). Contact toxicity, antifeedant activity, and oviposition preference of osthole against agricultural pests. Insects.

[98] Idrees A., Afzal A., Chohan T.A., Hayat S., Qadir Z.A., Gaafar A.R.Z., Zuan A.T.K., Li J. (2023). Laboratory evaluation of selected botanicals and insecticides against invasive *Spodoptera frugiperda* (Lepidoptera: Noctuidae). J. King Saud Univ. Sci..

[99] Zhang Z.B., Hu Q., Du X.G. (2022). Acaricidal of activity of matrine and osthole mixture against *Tetranychus urticae* Koch on Strawberry. Chin. J. Biol. Control.

[100] Zeng X.H., Chen S.H., Wang S.Z., Zhang L., Xu P., Guo Y.H. (2019). Study on the contact toxicity and stomach poisoning of four botanical pesticide against *Coptotermes formosanus*. Chin. J. Hyg. Insectic. Equip..

[101] Xia C.X., Li S.Q., Cai W.L., Yan X., Zhang H.Y. (2009). Insecticidal activity of osthole powder and its effect on enzyme activity of *Sitophilus zeamais*. Chin. Bull. Entomol..

[102] Meng L.L., Wang J.Z., Zhang Z.Y., Sun S.L., Zhang M.Z., Zhang P.F., Ding B.L., Pu Y.Y. (2010). Stomach action of osthole and its effect on two enzyme activities of *Plutella xylostella* larvae. J. Beijing Univ. Agric..

[103] Wang Z.Q., Kim J.R., Wang M., Shu S.H., Ahn Y.J. (2012). Larvicidal activity of *Cnidium monnieri* fruit coumarins and structurally related compounds against insecticide-susceptible and insecticide-resistant *Culex pipiens pallens* and *Aedes aegypti*. Pest Manag. Sci..

[104] Sun X.Y., Sun L.L., Qi H., Gao Q., Wang G.X., Wei N.N., Wang K. (2018). Antipruritic effect of natural coumarin osthole through selective inhibition of thermosensitive TRPV3 channel in the skin. Mol. Pharmacol..

[105] Torres K.V., Pantke S., Rudolf D., Eberhardt M.M., Leffler A. (2022). The coumarin osthole is a non-electrophilic agonist of TRPA1. Neurosci. Lett..

[106] Wang P.P., Fan Z.J., Wei W.P., Yang C.S., Wang Y., Shen X., Yan X., Zhou Z.H. (2023). Biosynthesis of the plant coumarin osthole by engineered *Saccharomyces cerevisiae*. ACS Synth. Biol..

[107] Wang S., Xie Y., Huo Y.W., Li Y., Abel P.W., Jiang H.H., Zou X.H., Jiao H.Z., Kuang X.L., Wolff D.W. (2020). Airway relaxation mechanisms and structural basis of osthole for improving lung function in asthma. Sci. Signal..

[108] Park J., Shin S., Bu Y., Choi H.Y., Lee K. (2024). Vasorelaxant and blood pressure-lowering effects of *Cnidium monnieri* fruit ethanol extract in sprague dawley and spontaneously hypertensive rats. Int. J. Mol. Sci..

[109] Liu G.L., Hao B., Liu S.P., Wang G.X. (2012). Synthesis and anthelmintic activity of osthol analogs against *Dactylogyrus intermedius* in goldfish. Eur. J. Med. Chem..

[110] Yin Q., Yan H., Zhang Y.Q., Wang Y.G., Zhang G.J., He Y.X., Zhang W.H. (2013). Palladium-catalyzed synthesis of 8-allyl or 8-prenylcoumarins by using organotin reagents as multicoupling nucleophiles. Appl. Organomet. Chem..

[111] Konrádová D., Kozubíková H., Dolezal K., Pospísil J. (2017). Microwave-assisted synthesis of phenylpropanoids and coumarins: Total synthesis of osthol. Eur. J. Org. Chem..

[112] Schmidt B., Riemer M. (2016). Synthesis of allyl- and prenylcoumarins via microwave-promoted tandem claisen rearrangement/wittig olefination. Synthesis.

[113] Gulías M., Marcos-Atanes D., Mascareñas J.L., Font M. (2019). Practical, large-scale preparation of benzoxepines and coumarins through Rhodium(III)-catalyzed C-H activation/annulation reactions. Org. Process Res. Dev..

[114] Dai P., Wang Q.Q., Teng P., Jiao J., Li Y.F., Xia Q., Zhang W.H. (2024). Design, synthesis, antifungal activity, and 3D-QASR of novel oxime ether-containing coumarin derivatives as potential fungicides. J. Agric. Food Chem..

[115] Ma H.N., Wang K.H., Wang B.B., Wang Z.W., Liu Y.X., Wang Q.M. (2024). Design, synthesis, and biological activities of novel coumarin derivatives as pesticide candidates. J. Agric. Food Chem..

[116] Zhang M.Z., Zhang R.R., Wang J.Q., Yu X., Zhang Y.L., Wang Q.Q., Zhang W.H. (2016). Microwave-promoted synthesis of novel fused osthole analogues. Chin. J. Chem..

[117] Zhang M.Z., Zhang R.R., Wang J.Q., Yu X., Zhang Y.L., Wang Q.Q., Zhang W.H. (2016). Microwave-assisted synthesis and antifungal activity of novel fused Osthole derivatives. Eur. J. Med. Chem..

[118] Zhang S.G., Liang C.G., Sun Y.Q., Teng P., Wang J.Q., Zhang W.H. (2019). Design, synthesis and antifungal activities of novel pyrrole- and pyrazole-substituted coumarin derivatives. Mol. Divers..

[119] Li S.C., Lv M., Sun Z.Q., Hao M., Xu H. (2021). Optimization of osthole in the lactone ring: Structural elucidation, pesticidal activities, and control efficiency of osthole ester derivatives. J. Agric. Food Chem..

[120] Yang H.Y., Hsu Y.F., Chiu P.T., Ho S.J., Wang C.H., Chi C.C., Huang Y.H., Lee C.F., Li Y.S., Ou G. (2013). Anti-cancer activity of an osthole derivative, NBM-T-BMX-OS01: Targeting vascular endothelial growth factor receptor signaling and angiogenesis. PLoS ONE.

[121] Pai J.T., Hsu C.Y., Hua K.T., Yu S.Y., Huang C.Y., Chen C.N., Liao C.H., Weng M.S. (2015). NBM-T-BBX-OS01, semisynthesized from osthole, induced G1 growth arrest through HDAC6 inhibition in lung cancer cells. Molecules.

[122] Zhang L., Wu Y.H., Yang G.X., Gan H.X., Sang D.Y., Zhou J.Y., Su L., Wang R., Ma L. (2020). Design, synthesis and biological evaluation of novel osthole-based derivatives as potential neuroprotective agents. Bioorg. Med. Chem. Lett..

[123] Ren Z.L., Lv M., Xu H. (2022). Osthole: Synthesis, structural modifications, and biological properties. Mini-Rev. Med. Chem..

[124] Hao M., Jiang L.L., Lv M., Ding H.X., Zhou Y.M., Xu H. (2024). Plant natural product-based pesticides in crop protection: Semi-synthesis, mono-crystal structures and agrochemical activities of osthole ester derivatives, and study of their toxicology against *Tetranychus cinnabarinus* (Boisduval). Pest Manag. Sci..

[125] Hao M., Lv M., Zhou L., Li H.J., Xu J.W., Xu H. (2022). Construction, pesticidal activities, control effects, and detoxification enzyme activities of osthole ester/amide derivatives. J. Agric. Food Chem..

[126] Ren Z.L., Lv M., Sun Z.Q., Li T.Z., Zhang S.Y., Xu H. (2021). Regioselective hemisynthesis and insecticidal activity of C8-hydrazones/acylhydrazones/sulfonylhydrazones coumarin-type derivatives of osthole. Bioorg. Med. Chem. Lett..

[127] Huang W.J., Chen C.C., Chao S.W., Lee S.S., Hsu F.L., Lu Y.L., Hung M.F., Chang C.I. (2010). Synthesis of *N*-hydroxycinnamides capped with a naturally occurring moiety as inhibitors of histone deacetylase. Chemmedchem.

[128] Huang W.J., Chen C.C., Chao S.W., Yu C.C., Yang C.Y., Guh J.H., Lin Y.C., Kuo C.I., Yang P., Chang C.I. (2011). Synthesis and evaluation of aliphatic-chain hydroxamates capped with osthole derivatives as histone deacetylase inhibitors. Eur. J. Med. Chem..

[129] Ren Z.L., Lv M., Li T.Z., Hao M., Li S.C., Xu H. (2020). Construction of oxime ester derivatives of osthole from *Cnidium monnieri*, and evaluation of their agricultural activities and control efficiency. Pest Manag. Sci..

[130] Shan X.J., Lv M., Wang J.R., Qin Y.J., Xu H. (2022). Acaricidal and insecticidal efficacy of new esters derivatives of a natural coumarin osthole. Ind. Crops Prod..

[131] Farooq S., Alharthi F.A., Alsalme A., Hussain A., Dar B.A., Hamid A., Koul S. (2020). Dihydropyrimidinones: Efficient one-pot green synthesis using Montmorillonite-KSF and evaluation of their cytotoxic activity. RSC Adv..

[132] You L.S., An R., Wang X.H., Li Y.M. (2010). Discovery of novel osthole derivatives as potential anti-breast cancer treatment. Bioorg. Med. Chem. Lett..

